# Perinatal intervention strategies providing food with micronutrients to pregnant and breastfeeding women in low‐ and middle‐income countries: A scoping review

**DOI:** 10.1111/mcn.13681

**Published:** 2024-07-01

**Authors:** Christine M. McDonald, K. Ryan Wessells, Christine P. Stewart, Kathryn G. Dewey, Saskia de Pee, Ritu Rana, Hajra Hafeez‐ur‐Rehman, Martin N. Mwangi, Sonja Y. Hess

**Affiliations:** ^1^ Departments of Pediatrics, and Epidemiology & Biostatistics University of California San Francisco California USA; ^2^ Institute for Global Nutrition and Department of Nutrition University of California Davis California USA; ^3^ Nutrition Division World Food Programme HQ Rome Italy; ^4^ Micronutrient Forum Washington, DC USA

**Keywords:** antenatal nutrition, balanced energy protein, lactation, LNS, maternal nutrition, perinatal nutrition, pregnancy, undernutrition

## Abstract

In resource‐constrained settings, pregnant and breastfeeding women and girls (PBW/G) are particularly vulnerable to undernutrition. Micronutrient‐fortified balanced energy protein (BEP) supplementation may be provided to boost maternal nutritional status and improve birth and infant outcomes. We conducted a scoping review of the published literature to determine the impact of BEP and other related nutrition interventions that provided fortified food or cash along with a minimum of 3 micronutrients on maternal, birth, and infant/child outcomes in low‐ and middle‐income countries. We conducted a PubMed search using pre‐defined keywords and controlled vocabulary search terms. All titles and abstracts were reviewed for eligibility by two independent reviewers, and data were extracted according to outcome type. We identified 149 eligible research articles that reported on a total of 21 trials and/or programme evaluations which assessed the health impact of one or more products (fortified lipid‐based nutrient supplement [LNS, *n* = 12], fortified blended flours [*n* = 5], milk‐based beverages [*n* = 2], and local food/snacks [*n* = 3]) that provided 118–750 kcal/day and varying levels of protein and micronutrients. Only one of these programme evaluations assessed the impact of the provision of cash and fortified food. Effects on maternal outcomes such as gestational weight gain and duration of gestation were promising but inconsistent. Birth outcomes were reported in 15 studies, and the effects on birthweight and birth length were generally positive. Seven studies demonstrated sustained benefits on infant and child growth out of the 15 studies that reported at least one of these outcomes, although data were sparse. Additional research is needed to investigate issues of dose, cost‐effectiveness, and incorporation into multi‐component interventions.

## Key messages

- There is great heterogeneity in the types of BEP provided to PBW/G in studies and programme evaluations, which impedes conclusions about comparative effectiveness.
- Many trials of antenatal nutrition interventions providing specialized nutritious food or food plus supplementation with at least three micronutrients demonstrated positive effects on birthweight and length. There were also some indications of benefit on gestational weight gain, duration of gestation, and infant/child growth; however, data were sparse, and results were inconsistent.
- No obvious dose–response relationship was observed between the amount of LNS provided and effects on maternal, birth or infant outcomes. Further research on this issue is needed.
- Evidence on the effects of cash interventions plus supplementation with at least 3 micronutrients was particularly limited. Additional programmatic assessments are needed before conclusions can be drawn about the effectiveness of this approach.
- Operational research is needed to compare product types, estimate cost‐effectiveness, and evaluate multi‐component packages under programme conditions.

## Graphical abstract

There is great heterogeneity in the types of balanced energy protein (BEP) supplementation provided to pregnant and breastfeeding women in studies and programme evaluations. Many trials demonstrated positive effects of BEP on birth outcomes; however, data on gestational weight gain, duration of gestation, and infant/child growth are sparse.

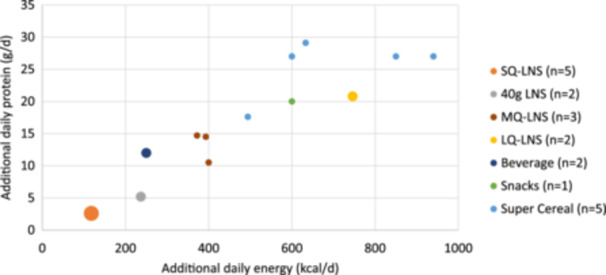

## 1 INTRODUCTION

The global burden of maternal undernutrition remains unacceptably high. In 2015, 14.3% of women 20–49 years of age living in low‐ and middle‐income countries (LMIC) had a low body mass index (BMI) and the prevalence of anaemia among women 15‐49 years of age was nearly 50% (Victora et al., 2021). Globally, it is estimated that 69% of women of reproductive age suffer from at least one micronutrient deficiency (Stevens et al., 2022). However, within a country, these estimates vary widely according to geography and socioeconomic status. Pregnant and breastfeeding women and girls (PBW/G) are particularly vulnerable to undernutrition given their elevated nutrient requirements and the various socioeconomic, availability, and other factors that may limit their dietary intake of nutrient‐dense foods in resource‐constrained settings. Depending on the stage of pregnancy and lactation, PBW/G require an additional 85–675 kcal/day and 1–31 g protein/day (FAO et al., 2004; WHO & FAO, 2004). Dietary micronutrient (vitamins and minerals) requirements are also increased by up to 50% during this vulnerable period, although physiologic adjustments may improve the absorption or utilization of selected micronutrients to compensate for increased demand (Bourassa et al., 2019; Kominiarek & Rajan, 2016). Various vitamins and minerals play essential roles in distinct phases of foetal development, encompassing implantation and vascularization of the placenta, offspring morphogenesis and organogenesis, neurological development and accumulation of foetal nutrient stores, as well as tissue deposition and body composition (Gernand et al., 2016). Suboptimal nutrition during these critically important life stages can impair foetal growth and development through infancy and early childhood and increase the risk of several adverse health outcomes for both the mother and the child. Interventions to improve the nutritional status of PBW/G have the potential to yield both short‐ and long‐term benefits and break the intergenerational cycle of undernutrition (Hofmeyr et al., 2023; Keats, Das, et al., 2021).

The World Health Organization (WHO) currently recommends that antenatal balanced energy and protein (BEP) supplementation be provided in settings where the prevalence of low BMI among women of reproductive age is 20% or more (WHO, 2016). Relatedly, in some humanitarian contexts, BEP supplementation, most commonly in the form of fortified blended food (FBF), may be provided to vulnerable PBW/G to prevent and/or treat undernutrition. However, uncertainty remains regarding the definition and optimal composition of BEP supplements. An Expert Consultation (Members of an Expert Consultation on Nutritious Food Supplements for Pregnant and Lactating Women, 2019) convened by the Bill & Melinda Gates Foundation in 2016 proposed a recommended formulation for BEP that would provide 250–500 kcal per daily serving and include 14–18 g protein and between approximately 1 EAR and 1 RDA of multiple micronutrients. In practice, however, many studies and programmes have provided supplemental food containing anywhere from 120 to nearly 1000 kcal/day. In addition to BEP, antenatal multiple micronutrient supplements (MMS) that include iron, folic acid, and other essential micronutrients are recommended by WHO in the context of rigorous research (WHO, 2020), and there is growing consensus that MMS during pregnancy are more beneficial than supplementation with iron and folic acid (IFA) alone (Gomes et al., 2022; Smith et al., 2017; Sudfeld & Smith, 2019). As such, the approach to providing BEP while meeting micronutrient requirements may vary in different contexts. In some settings, lipid‐based nutrient supplements (LNS) designed specifically for PBW/G may be provided as a single product. In others, FBF or non‐fortified food rations, snacks, or cash may be provided in combination with MMS. Questions remain regarding the effectiveness of these different approaches, and consensus has not been reached regarding the optimal dose and duration of supplementation, appropriate targeting and graduation criteria, and best modes/platforms of delivery.

To synthesize the available evidence, provide guidance for programme implementation, and refine future research priorities on nutritional interventions during pregnancy and lactation, we conducted a scoping review of the published literature on BEP, other related nutrition or cash interventions provided during pregnancy and/or lactation for the prevention and/or treatment of undernutrition and the prevention of poor birth outcomes in LMIC. Recognizing the widely accepted view that MMS (i.e. beyond iron and folic acid) are essential for a healthy pregnancy (Gomes et al., 2022; Smith et al., 2017), we were specifically interested in interventions that provided specialized nutritious foods fortified with at least three micronutrients, or unfortified food or cash provided with a supplement containing at least three micronutrients. A scoping review was determined to be the most suitable tool to determine the scope of the evidence, identify knowledge gaps, and inform practice given the expected heterogeneity in study interventions and study design in this field (Munn et al., 2018). Our objective was to review: (1) assessments of the effects of interventions on maternal, infant and/or child nutrition and health outcomes, (2) assessments on utilization and acceptability, (3) review articles, and (4) ongoing research regarding the aforementioned interventions.

## 2 METHODS

This scoping review focused on published research trials and programme assessments of interventions that aimed to improve the nutritional status of PBW/G in LMICs through the provision of specialized nutritious food fortified with at least three micronutrients or unfortified foods or cash provided with a minimum of three micronutrients in the form of a supplement (Table 1). There were no inclusion criteria set regarding the minimum amount of energy  and/or protein or the amount of cash provided. Micronutrients could have been provided either as part of the food (e.g., fortified food or LNS), as MMS or as a micronutrient powder (MNP). The primary outcomes of interest included women's, infants' and children's nutrition and health outcomes. A secondary objective was to describe quantitative and qualitative assessments of acceptability, adherence, and utilization of the interventions provided. In addition, we conducted a review of reviews to put the present scoping review into the larger perspective of scientific evidence and programme experiences. Because not all food‐based interventions included ≥3 micronutrients in the past, this eligibility criterion was relaxed for the summary of reviews. Lastly, we strove to summarize ongoing research to highlight current priorities, identify evidence gaps, and underscore future research needs.

**Table 1:** PICOS elements to define eligibility of studies for inclusion in the scoping review.

	Quantitative impact assessments on health and nutrition outcomes	Acceptability, adherence and utilization of interventions provided
Population of interest	PBW/G in LMIC	PBW/G in LMIC
Interventions	Provision of food, food vouchers or cash along with, or incorporating, ≥3 micronutrients*	Provision of food, food vouchers or cash along with, or incorporating, ≥3 micronutrients
Control	Control group receiving no food or cash but the same micronutrients, other types of control group, and the non‐intervention control group (i.e. standard of care)	With or without a control group
Outcomes	Gestational weight gain, birthweight, LBW, preterm delivery, gestational age at birth, SGA; anthropometric outcomes at 3, 6 and >6 mo, mortality, anaemia	Reported or observed adherence, acceptability
Study design	Randomized intervention trial, quasiexperimental, time series with multiple measurements before and after the introduction of the intervention	Same as *quantitative assessment*, plus observational qualitative trials

* Studies that compared multiple food supplements without a control or non‐intervention group were considered, given their relevancy for decision‐makers and programme implementers, and summarized in Supporting Information File 1: Results Section 2.

A protocol describing the methods used for the present scoping review was published online (Hess et al., 2023). Briefly, to identify relevant published scientific articles, we conducted a PubMed search using pre‐defined keywords and controlled vocabulary search terms (Supporting Information File 2: Table S1). The search strategy used the following approach: (A *OR* (B *AND* C)) *AND* D *AND* E. Search terms describing the intervention and control consisted of various terms describing fortified products (domain A), either food‐based or social assistance interventions (i.e., cash, vouchers, coupons) (domain B), or micronutrients (domain C). Terms describing the population of interest included terms used to describe PBW/G (domain D) and country/region (domain E). The search terms in domains A, B, and D were used for searches in the title and abstract only. The search terms for domain C (i.e., micronutrients) and E (i.e., LMIC settings) were expanded to all fields as this information may have been described only in the methods section of published articles. We did not define language or time restrictions when implementing the search. However, only articles published in English were included in the screening review process. Review articles of any type were considered eligible if published within the past 10 years.

We further identified potential studies for inclusion in the scoping review by cross‐referencing with recent systematic reviews and meta‐analyses (Das et al., 2018; Dewey, Stewart, et al., 2021; Imdad & Bhutta, 2011; Ota et al., 2015; Stevens et al., 2015), and the list of BEP studies included in the report of the Expert Consultation on the nutritional composition of a food supplement for pregnant and lactating women (Members of an Expert Consultation on Nutritious Food Supplements for Pregnant and Lactating women, 2019). During this process, we identified a few additional studies, which led us to update the search terms and run the final PubMed search on 31 August 2023 (Hess et al., 2023). A similar search term list was used to search the trial registries of the WHO (International Clinical Trials Registry Platform) and ClinicalTrials.gov (Supporting Information File 2: Table S2). For both searches, LMIC settings were screened manually.

All titles and abstracts were reviewed for eligibility by two independent reviewers in the Covidence systematic review software (Veritas Health Innovation, Melbourne, Australia). Conditions for the screening of titles and abstracts were defined such that each article was reviewed by at least one of the core investigators (CMM, KRW or SYH) and only once by a research assistant. During the screening of titles and abstracts, the location of the study was verified as LMIC with the World Bank classification (World Bank, 2023). Study protocol articles were not considered for inclusion in the outcome assessment part of the scoping review, even if all eligibility criteria were met. However, they were considered in the section on ongoing research. Any disagreements were resolved by an additional core investigator (CMM, KRW or SYH) while remaining blinded to the previous votes. During the full‐text review, each article was reviewed in more detail by one of the core investigators (CMM, KRW or SYH) to determine that the study design met the pre‐defined eligibility criteria (Hess et al., 2023). In case of uncertainties, consensus was sought through discussion among the three investigators.

Data were extracted according to outcome type (maternal, birth, infant/child) into tables in Excel by one of the core investigators (KRW). These detailed tables are available online (Hess et al., 2023). Due to the inconsistency in how the main outcome variables were defined, definitions were included along with the results in the extraction tables. Only data on the main effects of the intervention were extracted; the results of any subgroup analyses were not. The extracted data were then verified by a second core investigator (CMM) by consulting the primary articles. Summary tables were then generated to synthesize the effects of the intervention on related outcomes (e.g., gestational age at birth and preterm birth) evaluated in each study as having a positive/beneficial effect (green shading), a negative/detrimental effect (pink shading), mixed effects (yellow shading) or a null/nonsignificant effect (grey shading) on at least one of the related outcome variables. P values < 0.05 were considered statistically significant for the purposes of the synthesis activity. Critical appraisal was not performed.

## 3 RESULTS

### 3.1 Overview of included studies

**Figure 1:**
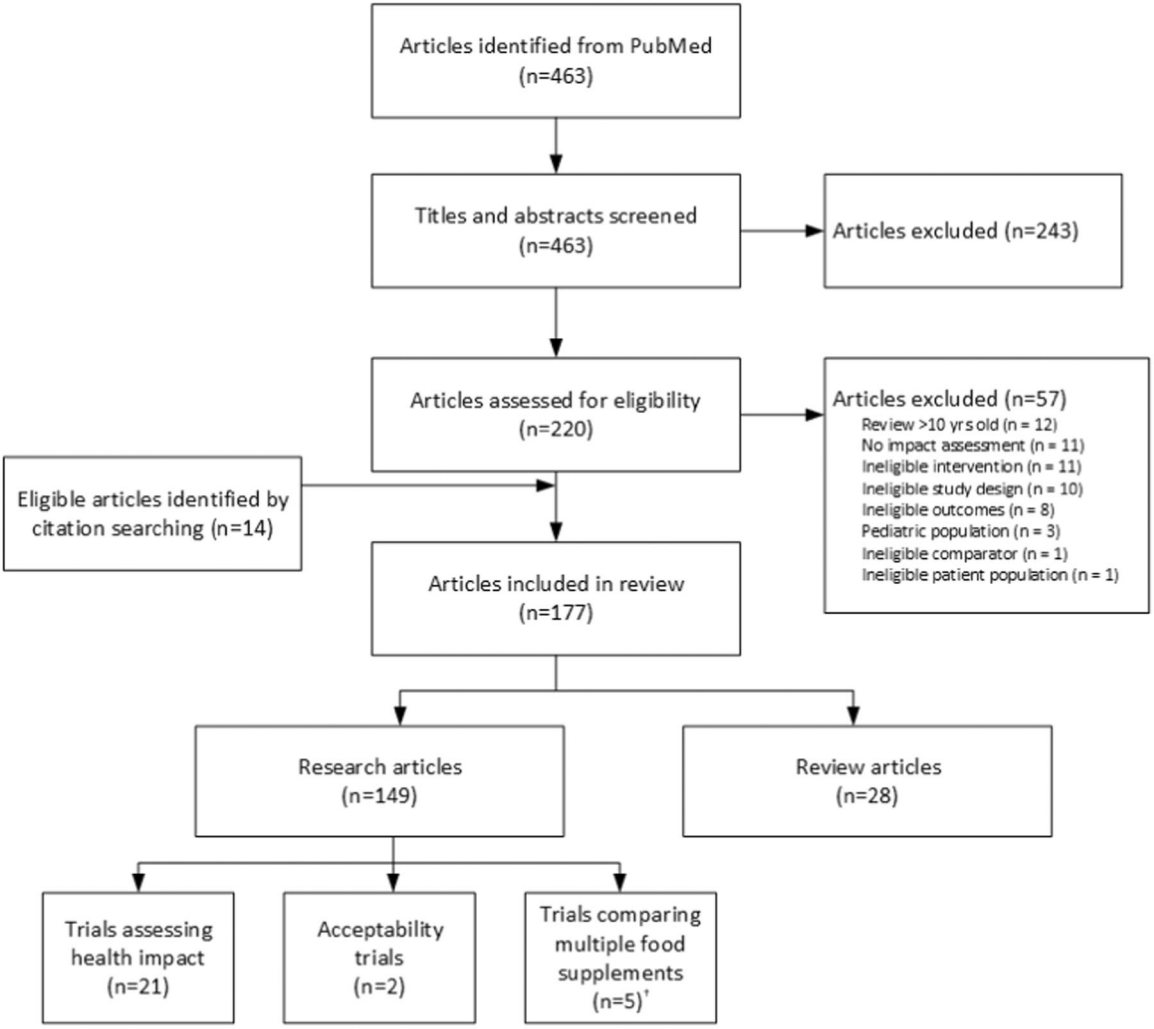
PRISMA flowchart of identified and included articles. ^†^Trials comparing multiple food supplements are summarized in the online supplemental results Section 1.

**Figure 2:**
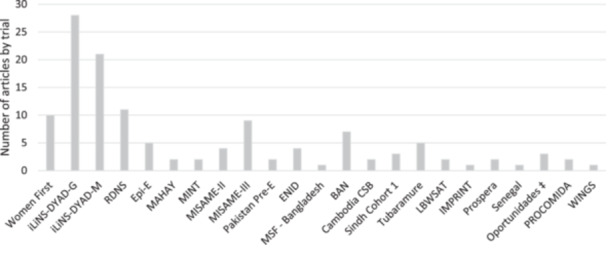
Number of articles reporting on health outcomes by intervention trial^†^. ^†^Five articles reported on outcomes collected in the two trials. ^‡^Several additional articles reported re‐analyses and other aspects of the programme ‘cash‐transfer Progresa–Oportunidades–Prospera’.

**Figure 3:**
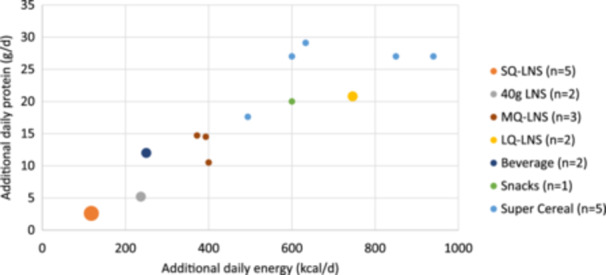
Additional daily energy and protein content by food type provided in intervention trials and programme evaluations^†^. ^†^The multi‐component intervention strategy of the WINGS trial not shown because the amount of energy and protein content depended on women's antenatal period and BMI. Acceptability studies and trials comparing multiple foods without a control group are also not shown. ^‡^In study arms 1 and 2 of Women First, women with BMI < 20 kg/m^2^ or inadequate gestational weight gain received an additional 55 g LNS per day. PROCOMIDA provided an additional family ration of rice and beans, not added to the graph. ^§^Oportunidades provided cash and a fortified milk‐based beverage. ^¶^Energy and protein content shown are for a daily ration of CSB, CSB+ and WSB+, including sugar and oil provided to the PBW/G. Cambodia CSB, Tubaramure, LBWSAT and PROCOMIDA also provided family ration, not added in the graph. ^††^BAN study provided weekly maize flour for family sharing, not added to the graph.

We identified 463 articles through PubMed (Figure 1) and excluded 243 after screening titles and abstracts. An additional 57 articles were excluded during the full‐text review. With an additional 14 eligible articles identified through citation searching, we included a total of 177 articles, of which 149 were research articles reporting primary or secondary results of intervention trials or programme evaluations, and 28 were review articles. The 149 research articles reported on a total of 28 studies (Table 2; Supporting Information File 2: Table S3). Twenty‐one trials assessed the nutrition or health impact of an eligible intervention versus a control and thus met the pre‐defined eligibility criteria (Figure 1). Four of these trials contributed over 50% of the articles identified (Figure 2). Among all reviewed studies, SQ‐LNS trials provided the lowest amount of supplemental food (118 kcal and 2.6 g protein/day) compared with LQ‐LNS (746 kcal and 20.8 g protein/day) and FBF (600–700 kcal/day and 20–29 g protein/day from the cereal distributed with fortified oil, which provided an additional 90–180 kcal/day) (Figure 3). Various numbers and amounts of micronutrients were added to the daily ration provided. Of note, the LBWSAT study in Nepal included an intervention group that provided cash (Saville et al., 2018). However, because participants assigned to this group did not also receive micronutrients, data were not extracted for analysis. The Oportunidades study in Mexico was the only included study that provided cash as part of a multi‐component intervention (LeRoy et al., 2018). A more detailed overview of the included studies is provided in Supporting Information File 1: Results section 1.

**Table 2:** Overview of study designs, interventions and populations with a focus on perinatal interventions.†

Study name	Country/ies (years of study implementation)	Study design	Study arms†	Period of perinatal intervention†	Type of food or cash‐based perinatal intervention†	Nutrition composition of food‐based intervention†	Target population for perinatal intervention†	Total *N* enrolled†	Primary reference
**LNS studies**									
Women First	Democratic Republic of the Congo, Guatemala, India, Pakistan (2013–2014)	RCT; individually randomized, unmasked, multisite	1 = Arm 1 (pre‐conception SQ‐LNS);2 = Arm 2 (SQ‐LNS at 12 weeks gestation); 3 = Control (SOC)	Pre‐conception and/or pregnancy	20 g LNS‐PBW/day; women with BMI < 20 kg/m^2^ or inadequate gestational weight gain in study arms 1 and 2 received additional 55 g LNS‐PBW/day	118 kcal, 2.6 g protein, MMN; second LNS‐PBW in study arms 1 and 2 provided 300 kcal, 11.2 g protein, no additional MMN	16–35 y; pre‐conception or 12–14 wk gestation	7387	Hambidge et al. (2019)
iLiNS‐DYAD‐G	Ghana (2009–2011)	RCT; individually randomized, partially double‐blind	1 = SQ‐LNS; 2 = MMS; 3 = Control (IFA)	Pregnancy and lactation	20 g LNS‐PBW/day	118 kcal, 2.6 g protein, MMN	≥18 y; <20 wk gestation	1320	Adu‐Afarwuah et al. (2017)
iLiNS‐DYAD‐M	Malawi (2011–2015)	RCT; individually randomized, partially double‐blind	1 = SQ‐LNS; 2 = MMS; 3 = Control (IFA)	Pregnancy and lactation	20 g LNS‐PBW/day	118 kcal, 2.6 g protein, MMN	≥15 y; <20 wk gestation	1391	Ashorn et al. (2015)
RDNS	Bangladesh (2011–2015)	RCT; cluster randomized, partially blind	1 = SQ‐LNS‐PBW, SQ‐LNS‐C; 2 = IFA, SQ‐LNS‐C; 3 = IFA, MNP‐C; 4 = Control (IFA)	Pregnancy and lactation	20 g LNS‐PBW/day	118 kcal, 2.6 g protein, MMN	≤20 wk gestation	4011	Matias et al. (2016)
Epi‐E	Niger (2015–2019)	RCT; cluster randomized, embedded in a vaccine trial, partially blind	1 = 40g‐LNS; 2 = MMS; 3 = Control (IFA)	Pregnancy	40 g LNS‐PBW/day	237 kcal, 5.2 g protein, MMN	<30 wk gestation	3332	Isanaka et al. (2021)
MAHAY	Madagascar (2014–2016)	RCT; cluster randomized, unmasked	1 = T1 (nutr counselling); 2 = T2 (SQ‐LNS‐C + nutr counselling); 3 = T3 (SQ‐LNS‐C and 40g‐LNS‐PBW + nutr counselling); 4 = T4 (early stimulation + nutr counselling); 5 = Control (SOC)	Pregnancy and lactation	40 g LNS‐PBW/day	235 kcal, 5.2 g protein, MMN	Pregnant women and mothers of 0–24 old children	3738 mothers; 1248 pregnant women	Galasso et al. (2019)
MINT	Nepal (2017–ongoing)	Acceptability study	1 = BEP supplement; 2 = Control (SOC)	Pregnancy and lactation	72 g LNS‐PBW/day; 75 g fortified vanilla biscuits/day	389 kcal, 14.5 g protein, MMN;375 kcal, 16.5 g protein, MMN	15–40 y; pregnant	40	Lama, et al. (2022)
MISAME‐II	Burkina Faso (2006–2008)	RCT; individually randomized, unmasked	1 = MQ‐LNS; 2 = Control (MMS)	Pregnancy	72 g LNS‐PBW/day	372 kcal, 14.7 g protein, MMN	Pregnant	1296	Huybregts et al. (2009)
MISAME‐III	Burkina Faso (2019–2021)	RCT; individually randomized, unmasked	1 = prenatal MQ‐LNS + IFA; 2 = postnatal MQ‐LNS + IFA; 3 = pre‐ and postnatal MQ‐LNS + IFA;4 = Control (IFA)	Pregnancy and/or lactation	72 g LNS‐PBW/day	393 kcal, 14.5 g protein, MMN	15–40 y <21 wk gestation	1897	de Kok et al. (2022)
Pakistan Pre‐E	Pakistan (2018–2020)	RCT; individually randomized, unmasked	1 = MQ‐LNS + IFA; 2 = Control (IFA)	Pregnancy	75 g LNS‐PBW/day	400 kcal, 10.5 g protein, MMN	15–35 y; pre‐ eclamptic; < recommended BMI for gestational age	60	Mohammad et al. (2022)
ENID	The Gambia (2010–2014)	RCT; individually randomized, partially blind	1 = MMS; 2 = LQ‐LNS + IFA; 3 = LQ‐LNS + MMS; 4 = Control (IFA)	Pregnancy	140 g LNS‐PBW/day	746 kcal, 20.8 g protein; with IFA or MMN	18–45 y; <20 wk gestation	620	Johnson et al. (2017)
MSF‐Bangladesh	Bangladesh (2011)	Acceptability study	1 = LQ‐LNS (RUTF) No control group	Pregnancy and lactation	1–3 packs Plumpy'nut/day	Not available	PBW with MUAC <210 mm or the presence of severe nutritional oedema grade three	248	Ali et al. (2015)
BAN	Malawi (2004–2010)	RCT; factorial design, individually randomized, unmasked	1 = HAART;2 = LQ‐LNS; 3 = HAART + LQ‐LNS; 4 = Control (SOC)	Lactation	140 g LNS/day; 2 kg/wk of maize flour for family sharing	746 kcal, 20.8 g protein, MMN	Lactating, HIV‐1‐infected; ≥14 y if married, ≥18 y if not married, with HIV‐uninfected exclusively breastfed infant	2369	Flax et al. (2012)
**Supercereal (CSB+ or WSB**+**) studies**									
Cambodia CSB	Cambodia (2011–2013)	RCT; cluster randomized, partially double‐blind	1 = CSB + ; 2 = Control (SOC)	Pregnancy	200 g dry matter CSB + /day and 10 mL palmolein oil fortified with vitamin A and D/day	~760 kcal, ~27 g protein, MMN, plus ~90 kcal from daily oil ration	≥18 y; <13 wk gestation	547	Janmohamed et al. (2016)
Sindh Cohort 1	Pakistan (2014–2018)	RCT; cluster randomized, unmasked	1 = WSB + ; 2 = Control (SOC)	Pregnancy and lactation	165 g dry matter WSB + /day	~633 kcal, ~29.1 g protein, MMN	Pregnant	2030	Soofi et al. (2022)
Tubaramure	Burundi (2010–2012)	RCT; cluster randomized, unmasked	1 = T24 (CSB, pregnancy to 24 mo); 2 = T18 (CSB, pregnancy to 18 mo); 3 = TNFP (CSB, birth to 24 mo); 4 = Control (SOC)	Pregnancy and/or lactation	6 kg dry matter CSB + /mo and 0.6 L vegetable oil fortified with vitamin A and D/mo; and 12 kg CSB+ and 1.2 L oil for family sharing	~760 kcal, 27 g protein, MMN, plus ~180 kcal from daily oil ration to women	Pregnant women and mothers of <6 mo old children	2505 mothers	Leroy et al. (2018)
LBWSAT	Nepal (2013–2015)	RCT; cluster randomized, unmasked	1 = PLA only; 2 = PLA + Cash; 3 = PLA + WSB + ; 4 = Control (SOC)	Pregnancy	Cash: NPR750 ≈ US$7.5/mo; Food: 150 g dry matter CSB + /day; and 180 g CSB+ for family sharing	~600 kcal, >20 g protein, MMN	10–49 y	25,092	Saville et al. (2018)
**Other types of food supplements**									
IMPRINT	India (2018–2019)	RCT; individually randomized, unmasked	1 = Food Supplement (snack) + MMS; 2 = Control (SOC); both groups counselled on availability of IFA, Ca and vitamin D through national programme + BCC	Lactation	Food supplement in form of sweet or savoury snacks: choco energy bites, panjeeri, jeera crackers, nut mixtures, biscuits	600 kcal, 20 g protein; MMN supplements	Mothers of infants initiated to breastfeeding and enrolled < 7 days old	816	Taneja et al. (2021)
Prospera‡	Mexico (2005–2007)	RCT; cluster randomized, unmasked	1 = Nutrivida (fortified milk‐based beverage); 2 = MMS; 3 = MNP	Pregnancy and lactation	54 g Nutrivida (fortified milk‐based beverage)	250 kcal, 12 g protein, MMN	>18 y; <25 wk gestation	715	Neufeld et al. (2019)
Senegal	Senegal (not available)	Case–control, deuterium study	1 = Millet food supplement; 2 = Maize food supplement; 3 = Control (non‐supplemented)	Pregnancy and lactation	Fortified millet‐based food supplement; fortified corn‐based food supplement	Millet: 422 kcal/100 g dry matter; maize: 400 kcal/100 g dry matter	Pregnant women participating in governmental community nutrition project	133	Cisse et al. (2002)
**Food supplement provided as part of a bundled intervention**									
Oportunidades‡	Mexico (2002–2004)	Impact evaluation	1 = Intervention (cash transfers, fortified food targeted to PBW, children 6–23 mo and children with low weight 2–4 y); 2 = Control (comparison households)	Pregnancy and lactation	Cash: 32.5–41.3 USD/mo; Nutrivida (fortified milk‐based beverage): 52 g dry powder to consume as 202 g beverage/day	250 kcal, 12–15 g protein; MMN	Recipients of Oportunidades	733	Leroy et al. (2008)
PROCOMIDA	Guatemala (2011–2015)	RCT; cluster randomized, unmasked	1 = FFR + CSB; 2 = RFR + CSB; 3 = NFR + CSB; 4 = FFR + SQ‐LNS; 5 = FFR + MNP; 6 = Control (SOC)	Pregnancy and lactation	FFR and RFR: rice, beans and oil; ~130 g dry matter CSB/day; 20 g LNS/day	FFR: 269 kcal/family member/day; RFR: 152 kcal/family member/day; CSB: 494 kcal/day; LNS: 118 kcal/day	3−7 mo pregnant	3535	Olney et al. (2018)
WINGS	India (2017–2021)	RCT; individually randomized, unmasked	1 = Preconception and pregnancy and early childhood interventions§; 2 = Preconception interventions only§; 3 = Pregnancy and early childhood interventions only§; 4 = Control (SOC)	Preconception and/or pregnancy	Locally prepared foods (snacks, milk, egg); MMS and IFA	Level of kcal and protein depending on women's antenatal period and BMI§	18–30 y	4921	Taneja et al. (2022)
**Studies comparing multiple types of food supplements**									
INCAP	Guatemala (1969–1977)	Community RCT; cluster randomized, unmasked	1 = Atole; 2 = Control (Fresco)	Preconception, pregnancy and lactation	180 mL atole (corn‐based, protein‐rich supplement); 180 ml fresco (drink, caloric supplement)¶	Atole: ad lib, 163 kcal, 11 g protein/serving/day; Fresco: 59 kcal, 0 g protein/serving/day	Pregnant, breastfeeding or in formed unions/married without children	830	Delgado et al. (1982)
MINIMat	Bangladesh (2001–2009)	RCT; factorial design, individually randomized, partially blinded	1 = 30 mg IFA, Early Food; 2 = 60 mg IFA, Early Food; 3 = MMS, Early Food; 4 = 30 mg IFA, Usual Food; 5 = MMS, Usual Food; 6 = Control (60 mg IFA, Usual Food)	Pregnancy	Food supplement based on rice and roasted pulse powder, molasses, soybean oil	680 kcal, 18 g protein/day	<14 wk gestation	4436	Khan et al. (2011)
South Africa Ross	South Africa (1977)	RCT; individually randomized, unmasked	1 = Low bulk supplement; 2 = High bulk supplement; 3 = Zinc supplement; 4 = Control (no supplement)	Pregnancy	Low bulk supplement: fortified porridge based on skimmed milk, maize flour; High bulk supplement: fortified mixture of beans and maize	Low bulk supplement: 700 kcal, 8 g vegetable protein, 36 g animal‐source protein, MMN; High bulk supplement: 776 kcal, 36 g vegetable protein, MMN;	<20 wk gestation	127	Ross et al. (1985)
Mamachiponde	Malawi (2014–2016)	RCT; individually randomized, single‐blind	1 = LNS (RUSF); 2 = CSB+ with UNIMMAP; 3 = Control (CSB + , IFA)	Pregnancy	10 ×250 g bottles of RUSF; 5 kg of dry matter CSB+ in biweekly rations	RUSF: 920 kcal, 36 g protein/day; CSB + : 893 kcal, 33 g protein/day	>18 yr, pregnant, with moderate malnutrition (MUAC ≥ 20.6 and ≤23.0 cm)	1828	Callaghan‐Gillespie et al. (2017)
Sierra Leone RUSF	Sierra Leone (2017–2019)	RCT; individually randomized, unmasked	1 = LNS (RUSF) + IPTp, azithromycin + dx/tx vaginal dysbiosis; 2 = Control (CSB + , IFA, IPTp)	Pregnancy	100 g RUSF; 250 g of dry matter CSB+ and 25 g palmolein oil/day	RUSF: 520 kcal, 18 g protein, MMN; CSB + : 589 kcal, 18 g protein, MMN, plus ration for family sharing	>18 yr, pregnant, undernourished (MUAC ≤ 23.0 cm), fundal height <35 cm	1489	Hendrixson et al. (2021)

Abbreviations: BCC, behaviour change communication; BMI, body mass index; CSB, fortified corn–soy blend; CSB + , corn–soy blend fortified with updated vitamin and mineral premix; FFR, full family ration; HAART, highly active antiretroviral therapy; IFA, iron‐folic acid supplements; IPTp, intermittent preventive treatment of malaria for pregnant women; LNS, lipid‐based nutrient supplements; LNS‐C; lipid‐based nutrient supplement for 6–24‐month old children; LNS‐PBW, lipid‐based nutrient supplement for pregnant and breastfeeding women; LQ, large‐quantity; MMN, multiple micronutrient; MNP, micronutrient powder; MNP‐C, micronutrient powder for 6–24‐month‐old children; MQ, medium‐quantity; MMS, multiple micronutrient supplement; NFR, no family ration; NPR, Nepalese rupees; nutr, nutrition; PBW, pregnant or breastfeeding women; PLA, participatory learning and action women's groups; RCT, randomized controlled trial; RFR, reduced family ration; SOC, standard of care; RUSF, ready‐to‐use supplemental food; RUTF, ready‐to‐use therapeutic food; SQ, small‐quantity; TNFP, no food during pregnancy; UNIMMAP, United Nations International Multiple Micronutrient Preparation; WSB + , wheat soy blend fortified with updated vitamin and mineral premix.

^†^ Overview table focuses on perinatal interventions targeting preconception, pregnant and/or lactation women; several studies also provided interventions to young children, which are not described in this table.

^‡^ The trials referred to here as ‘Prospera’ and ‘Oportunidades’ were evaluations of different aspects of the ‘cash‐transfer Progresa–Oportunidades–Prospera’ programme implemented by the Mexican government.

^§^ Multi‐component interventions of the WINGS trial: integrated and concurrent delivery of health, nutrition, water, sanitation and hygiene, and psychosocial care interventions (preconception, pregnancy, post‐partum and early childhood). Nutrition‐specific interventions included IFA, MMN, and food supplements (locally prepared snacks, eggs or milk). Pre‐conception: 500 kcal and 6–10 g protein/day for women with BMI 16–18.5 kg/m^2^ and twice the amount for women with BMI < 16 kg/m^2^. One egg or milk (180 mL) containing 70 kcal and 6 g protein for women with BMI < 21 kg/kg/m^2^ 6 days a week; Pregnancy: 210 kcal, 2 g protein in second trimester, 400 kcal, 21 g protein in third trimester. All women are also given milk (180 mL, 70 kcal, 6 g protein) 6 days a week throughout pregnancy. Additionally, women with BMI < 18.5 kg/m^2^ are given 500 kcal, 20 g protein in the form of a hot‐cooked meal as the first meal in the morning. Lactation: 500 kcal, 15 g protein as locally prepared snacks and milk (180 mL, 70 kcal, 6 g protein) 6 days a week.

^¶^ Data not analyzed according to original study design (atole vs. fresco), but by the total energy intake from the supplements across pregnancy (high vs. low).

### 3.2 Summary of maternal outcomes

Fifteen studies assessed the effects of food with micronutrients during the perinatal period on at least one maternal outcome (Table 3, Supporting Information File 3: Table S4).

**Table 3:** Summary of effects on maternal outcomes.†

Study name	Intervention groups	Gestational weight gain/inadequate weight gain	Maternal MUAC	Gestational age/preterm birth	Stillbirth	Maternal haemoglobin/anaemia
**LNS studies**						
Women First	1 = Arm 1 (pre‐conception SQ‐LNS); 2 = Arm 2 (SQ‐LNS at 12 weeks gestation); 3 = Control (SOC)					
iLiNS‐DYAD‐G	1 = SQ‐LNS; 2 = MMN; 3 = Control (IFA)					
iLiNS‐DYAD‐M	1 = SQ‐LNS; 2 = MMN; 3 = Control (IFA)					
RDNS	1 = SQ‐LNS‐PBW, SQ‐LNS‐C; 2 = IFA, SQ‐LNS‐C, 3 = IFA, MNP‐C, 4 = Control (IFA) [For outcomes, groups 2‐4 are combined as the control]					
MISAME‐II	1 = MQ‐LNS; 2 = Control (MMN)					
MISAME‐III	1 = prenatal MQ‐LNS + IFA; 2 = postnatal MQ‐LNS + IFA; 3 = pre‐ and postnatal MQ‐LNS + IFA; 4 = Control (IFA) [For outcomes, groups 2 and 4 are combined as the control]					
Pakistan Pre‐E	1 = MQ‐LNS + IFA; 2 = Control (IFA)					
ENID	1 = MMN; 2 = LQ‐LNS + IFA; 3 = LQ‐LNS + MMN; 4 = IFA					
BAN	1 = HAART; 2 = LQ‐LNS; 3 = HAART + LQ‐LNS; 4 = Control (SOC)					
**Supercereal (CSB+ or WSB** + **) studies**						
Cambodia CSB	1 = CSB + ; 2 = Control (SOC)					
Tubaramure	1 = T24 (CSB, pregnancy to 24 mo); 2 = T18 (CSB, pregnancy to 18 mo); 3 = TNFP (CSB, birth to 24 mo); 4 = Control (SOC)					
LBWSAT	1 = PLA only; 2 = PLA + Cash; 3 = PLA + WSB + ; 4 = Control (SOC)					
**Other types of food supplements**						
IMPRINT	1 = Food Supplement (snack) + MMN; 2 = Control (SOC); both groups counselled on availability of IFA, Ca and vitamin D through national programme + BCC					
Prospera	1= Nutrivida (fortified milk‐based beverage); 2 = MMN; 3 = MNP					
**Food supplement provided as part of a bundled intervention**						
WINGS	1 = Preconception and pregnancy and early childhood interventions; 2 = Preconception interventions only; 3 =Pregnancy and early childhood interventions only; 4 = Control (SOC)					

Abbreviations: BCC, behaviour change communication; CSB, fortified corn–soy blend; CSB + , corn–soy blend fortified with updated vitamin and mineral premix; HAART, highly active antiretroviral therapy; IFA, iron–folic acid supplements; LNS, lipid‐based nutrient supplements; LNS‐C, lipid‐based nutrient supplement for 6–24‐month‐old children; LNS‐PBW, lipid‐based nutrient supplement for pregnant and breastfeeding women; LQ, large quantity; MMN, multiple micronutrient; MNP, micronutrient powder; MNP‐C, micronutrient powder for 6–24‐month‐old children; MQ, medium quantity; PLA, participatory learning and action women's groups; SOC, standard of care; SQ, small quantity; TNFP, no food during pregnancy; WSB + , wheat soy blend fortified with updated vitamin and mineral premix

^†^ Green: positive/beneficial effect; pink: a negative/detrimental effect; yellow, mixed effects; grey: a null/nonsignificant effect on at least one of the related outcome variables; white, not measured or reported. *p* values < 0.05 were considered statistically significant for the purposes of the synthesis activity. Outcomes are as defined by investigators. Detailed results are available in Supporting Information File 3: Table S4.

#### 3.2.1 Effects of LNS on maternal outcomes

Among the six LNS studies that evaluated gestational weight gain as an outcome, two reported positive effects, and four did not detect any significant differences between study groups. In the Women First trial, gestational weight gain was approximately 0.7 kg greater among women who received 20 g LNS daily plus an additional 55 g daily among women who had a low BMI during the preconception period or inadequate gestational weight gain during pregnancy versus the SOC control group which did not receive any nutritional supplements provided by the study (*p* < 0.0015) (Hambidge et al., 2019). In the iLiNS‐DYAD‐G study, women who received SQ‐LNS were 15% less likely to have inadequate weight gain, as defined by the 2009 IOM guidelines, than women who received MMS (RR: 0.85 (95% CI: 0.74, 0.98; *p* = 0.02) (Adu‐Afarwuah, Lartey, Okronipa, Ashorn, Ashorn, et al., 2017).

Three of the four studies that provided SQ‐LNS during pregnancy did not detect any significant effects on gestational age/preterm birth (Adu‐Afarwuah et al., 2015; Ashorn, Alho, Ashorn, Cheung, Dewey, Harjunmaa, et al., 2015; Mridha et al., 2016) and results of the Women First trial were mixed (Hambidge et al., 2019). Of the studies that provided MQ‐LNS (~400 kcal/day), the MISAME‐III and Pakistan Pre‐E trials reported positive effects on gestational age at birth (de Kok et al., 2022; Mohammad et al., 2022); in contrast, the MISAME‐II trial did not observe any statistically significant differences between groups (Huybregts et al., 2009). Findings from the ENID trial are somewhat unique because births occurred at an earlier gestational age in the group that received LQ‐LNS (746 kcal/day) along with IFA alone versus other study groups (*p* = 0.02); however, there were no differences in gestational age between the group that received LQ‐LNS plus MMS versus other study groups (Johnson et al., 2017).

Of the five LNS studies that reported on stillbirth, four did not find any statistically significant differences across groups (Adu‐Afarwuah et al., 2015; de Kok et al., 2022; Huybregts et al., 2009; Mridha et al., 2016); however, it is likely that these studies may have been underpowered to detect significant differences in the rare outcome. The iLiNS‐DYAD‐M trial observed a stillbirth rate of 3.5% in the SQ‐LNS group versus 0.5% in the MMS group (*p* = 0.006) (Ashorn, Alho, Ashorn, Cheung, Dewey, Harjunmaa, et al., 2015).

The effects of LNS on maternal haemoglobin concentrations and/or anaemia were mixed among the seven studies that evaluated this outcome. When interpreting these results, it is important to consider that in many cases (e.g., iLiNS‐DYAD‐M, iLiNS‐DYAD‐G, RDNS), the dose of iron provided by the LNS was substantially lower (i.e., 20 mg) than the dose provided by the standard IFA supplement (60 mg). In other cases, the LNS groups received a higher iron dose than the control groups, either because the LNS group received an IFA supplement in addition to LNS (e.g., MISAME‐III, Pakistan Pre‐E), or the control group did not receive IFA supplementation (e.g., BAN). The Pakistan Pre‐E trial reported significantly higher haemoglobin concentrations post‐delivery among underweight women who received MQ‐LNS + IFA versus IFA during pregnancy (12.2 g/dL versus 11.4 g/dL; *p* = 0.001) (Sher et al., 2022). In contrast, the iLiNS‐DYAD‐G, iLiNS‐DYAD‐M and ENID trials reported lower haemoglobin concentrations among women who received LNS versus IFA (Adu‐Afarwuah, Lartey, Okronipa, Ashorn, Zeilani, et al., 2017; Jorgensen et al., 2018), and LNS versus IFA or MMS, respectively (Jobarteh et al., 2017).

#### 3.2.2 Effects of FBF on maternal outcomes

Maternal outcomes were not evaluated as consistently or comprehensively among the three trials that evaluated FBF (i.e. either CSB+ or WSB+). The Cambodia CSB trial reported a lower prevalence of inadequate gestational weight gain among women who received CSB + versus the SOC control (84% vs. 91%; *p* = 0.04) (Janmohamed, Karakochuk, Boungnasiri, Chapman, et al., 2016); however, the Tubaramure and LBWSAT trial either did not report (Leroy et al., 2018) or did not statistically compare this outcome (Saville et al., 2018). The Cambodia CSB study also reported a lower prevalence of preterm birth in the CSB+ versus the control group (2.1% vs. 7.1%; *p* = 0.03) (Janmohamed, Karakochuk, Boungnasiri, Chapman, et al., 2016), whereas the LBWSAT did not detect any significant differences between groups (Saville et al., 2018). The Cambodia CSB and Tubaramure trials also detected lower prevalences of anaemia among women who received CSB and IFA via routine antenatal services versus IFA provided via routine antenatal services alone (Janmohamed, Karakochuk, Boungnasiri, Chapman, et al., 2016; Leroy et al., 2016).

#### 3.2.3 Effects of other types of supplements and/or supplements provided as part of a bundled intervention on maternal outcomes

Very few maternal outcomes were evaluated among the two trials that provided other types of food supplements. The Prospera study did not detect any differences in gestational weight gain among women who received a fortified milk beverage versus MMS or MNPs (Neufeld et al., 2019). Likewise, it did not detect any differences in haemoglobin concentration or anaemia prevalence (Neufeld et al., 2019). The IMPRINT study, however, did report a mean haemoglobin concentration that was 0.37 g/dL higher among women in the intervention group who received a snack + MMS versus the SOC control group (Taneja et al., 2021).

Although the multi‐component design of the WINGS study precludes the effects of the food supplement from being isolated, gestational weight gain and mean haemoglobin concentrations were, respectively, 1.42 (1.15, 1.70) kg and 0.68 (0.56, 0.80) g/dL higher among women who received interventions in pregnancy versus those who did not, and 1.48 (1.1, 1.86) kg and 0.77 (0.59, 0.95) g/dL greater among women who received the comprehensive intervention in the preconception and pregnancy periods versus the SOC control group (Taneja et al., 2022).

### 3.3 Summary of birth outcomes

Fifteen studies assessed the effects of food with micronutrients during the antenatal period on at least one birth outcome (Table 4, Supporting Information File 4: Table S5).

**Table 4:** Summary of effects on birth outcomes.^†^

Study name	Intervention groups	Birthweight/WAZ	LBW (%)	SGA (%)	Birth length/LAZ/newborn stunting (%)	BMIZ, WLRAZ, ponderal index/low BMIZ, WLRAZ (%)	Birth head circ/HCAZ/low birth HCAZ	Birth MUAC (cm)	Neonatal mortality (≤7 d or ≤ 28 d)‡
**LNS studies**									
Women First	1 = Arm 1 (pre‐conception SQ‐LNS); 2 = Arm 2 (SQ‐LNS at 12 weeks gestation); 3 = Control (SOC)								
iLiNS‐DYAD‐G	1 = SQ‐LNS; 2 = MMN; 3 = IFA								
iLiNS‐DYAD‐M	1 = SQ‐LNS, 2 = MMN, 3 = Control (IFA)								
RDNS	1 = SQ‐LNS‐PLW, SQ‐LNS‐C; 2 = IFA, SQ‐LNS‐C; 3 = IFA, MNP‐C; 4 = Control (IFA) [For outcomes, groups 2‐4 are combined as the control]								
Epi‐E	1 = 40g‐LNS; 2 = MMN; 3 = Control (IFA)								
MISAME‐II	1 = MQ‐LNS; 2 = Control (MMN)								
MISAME‐III	1 = prenatal MQ‐LNS + IFA; 2 = postnatal MQ‐LNS + IFA; 3 = pre‐ and postnatal MQ‐LNS + IFA; 4 = Control (IFA) [For outcomes, groups 2 and 4 are combined as the control]								
Pakistan Pre‐E	1 = MQ‐LNS + IFA; 2 = Control (IFA)								
ENID	1 = MMN; 2 = LQ‐LNS + IFA; 3 = LQ‐LNS + MMN; 4 = Control (IFA)								
**Supercereal (CSB+ or WSB** + **) studies**									
Cambodia CSB	1 = CSB + ; 2 = Control (SOC)								
Sindh Cohort 1	1 = WSB + ; 2 = Control (SOC)								
LBWSAT	1 = PLA only; 2 = PLA + Cash; 3 = PLA + WSB + ; 4 = Control								
**Food supplement provided as part of a multi‐component intervention**									
Oportunidades	1 = Intervention (cash transfers, fortified food targeted to PLW, children 6‐23 mo and children with low weight 2‐4 y); Control = comparison households								
PROCOMIDA	1 = FFR + CSB;2 = RFR + CSB;3 = NFR + CSB;4 = FFR + SQ‐LNS;5 = FFR + MNP;6 = Control (SOC)								
WINGS	1 = Preconception and pregnancy and early childhood interventions; 2 = Preconception interventions only; 3 = Pregnancy and early childhood interventions only; 4 = Control (SOC)								

BMIZ, body mass index‐for‐age *z*‐score; circ, circumference; CSB, fortified corn–soy blend; CSB + , corn–soy blend fortified with updated vitamin and mineral premix; FFR, full family ration; IFA, iron‐folic acid supplements; HCAZ, head circumference‐for‐age *z*‐score; LAZ, length‐for‐age *z*‐score; LBW, low birthweight ( < 2500 g); LNS, lipid‐based nutrient supplement LNS‐C, lipid‐based nutrient supplement for 6–24‐month‐old children; LNS‐PBW, lipid‐based nutrient supplement for pregnant and breastfeeding women; LQ, large quantity; MMN, multiple micronutrients; MNP, micronutrient powder; MNP‐C, micronutrient powder for 6–24‐month‐old children; MQ, medium quantity; MUAC, mid‐upper arm circumference; NFR, no family ration; PLA, participatory learning and action women's groups; RFR, reduced family ration; SGA, small for gestational age; SOC, standard of care; SQ, small quantity; WAZ, weight‐for‐age *z*‐score; WLRAZ, weight to length ratio‐for‐age *z*‐score; WSB + , wheat soy blend fortified with updated vitamin and mineral premix.

^†^ Green: positive/beneficial effect; pink: a negative/detrimental effect; grey: a null/nonsignificant effect on at least one of the related outcome variables; white, not measured or reported. P values < 0.05 were considered statistically significant for the purposes of the synthesis activity. Outcomes are as defined by investigators. Detailed results are available in Supporting Information File 4: Table S5.

^‡^ Early neonatal mortality defined as deaths among live births within the first 7 completed days of life; neonatal mortality defined as deaths among live births within the first 28 completed days of life.

#### 3.3.1 Effects of LNS on birth outcomes

Three of the four SQ‐LNS studies that assessed birthweight and/or WAZ reported a positive effect on this outcome. Among these, birthweights were ~40–85 g higher in the SQ‐LNS versus comparison group(s) (Adu‐Afarwuah et al., 2015; Krebs et al., 2021; Mridha et al., 2016). The MISAME‐III and Pakistan Pre‐E studies provided MQ‐LNS during pregnancy and reported intervention effects of ~50 and ~120 g on birthweight, respectively (de Kok et al., 2022; Mohammad et al., 2022). However, the Epi‐E and MISAME‐II trials, which provided 40 and 75 g LNS during pregnancy, respectively, did not detect any significant group‐wise differences in weight at birth or shortly after birth (Bliznashka et al., 2022; Huybregts et al., 2009).

Of the 7 LNS studies that evaluated the prevalence of small‐for‐gestational‐age, positive effects of SQ‐LNS were observed in the Women First and RDNS trials (Hambidge et al., 2019; Mridha et al., 2016). The other five LNS studies (two SQ‐LNS, two MQ‐LNS, one LQ‐LNS) did not detect any significant differences in this outcome between groups (Adu‐Afarwuah et al., 2015; Ashorn, Alho, Ashorn, Cheung, Dewey, Harjunmaa, et al., 2015; de Kok et al., 2022; Huybregts et al., 2009; Johnson et al., 2017).

The majority of LNS studies that assessed birth length, LAZ and/or newborn stunting reported beneficial effects on at least one of these outcomes. Of the four studies that provided SQ‐LNS, the Women First and RDNS trials observed 31% and 17% reductions in newborn stunting in the preconception SQ‐LNS (10.0%) versus SOC control (14.2%) group (Hambidge et al., 2019) and SQ‐LNS (18.7%) versus IFA (22.6%) group (Mridha et al., 2016), respectively. However, the iLiNS‐DYAD‐G and iLiNS‐DYAD‐M trials did not detect any significant differences (Adu‐Afarwuah et al., 2015; Ashorn, Alho, Ashorn, Cheung, Dewey, Harjunmaa, et al., 2015). All three MQ‐LNS trials observed positive effects on birth length that ranged from ~0.2 to 0.6 cm (de Kok et al., 2022; Huybregts et al., 2009; Mohammad et al., 2022). The Epi‐E and ENID trials, which provided 40g‐LNS and LQ‐LNS, respectively, did not detect any significant intervention effects on LAZ (Bliznashka et al., 2022; Johnson et al., 2017).

Four of the seven LNS studies that evaluated some measure of wasting at birth (i.e., weight‐for‐length Z (WLZ) score, weight‐for‐length‐ratio‐for‐age Z (WLRAZ) score, BMI‐for‐age Z (BMIZ) score, ponderal index), detected benefits of LNS on this outcome. Three of the four studies reporting positive outcomes provided SQ‐LNS; effects of 40–72 g LNS/day were more variable.

Of the 8 LNS trials that evaluated head circumference at birth as an outcome, only the RDNS and Pre‐E trials reported positive effects of SQ‐LNS and MQ‐LNS, respectively (Mohammad et al., 2022; Mridha et al., 2016). The other 6 trials did not detect any significant intervention effects on this outcome (Adu‐Afarwuah et al., 2015; Ashorn, Alho, Ashorn, Cheung, Dewey, Harjunmaa, et al., 2015; de Kok et al., 2022; Hambidge et al., 2019; Huybregts et al., 2009; Johnson et al., 2017). Of the four trials that evaluated MUAC at birth as an outcome, the iLiNS‐DYAD‐G and MISAME‐III trials reported positive effects of SQ‐LNS of 0.2 and 0.09 cm, respectively (Adu‐Afarwuah et al., 2015; de Kok et al., 2022). The RDNS and MISAME‐II trials did not detect any significant differences between groups (Huybregts et al., 2009; Mridha et al., 2016).

As with stillbirth, neonatal mortality is a rare outcome, and most studies were likely underpowered to detect statistically significant differences in this outcome between groups. However, 5 LNS trials reported data on early neonatal or neonatal mortality rates. Four of these did not detect any significant differences between groups (Adu‐Afarwuah et al., 2015; Ashorn, Alho, Ashorn, Cheung, Dewey, Harjunmaa, et al., 2015; Huybregts et al., 2009; Mridha et al., 2016). However, the Women First trial observed a RR of neonatal mortality of 1.79 (1.08, 2.97) among infants whose mothers had received SQ‐LNS starting at 12 weeks gestation versus the SOC control group (Krebs et al., 2021).

#### 3.3.2 Effects of FBF on birth outcomes

In comparison to the LNS studies, substantially less data were available on the effects of FBF during pregnancy on birth outcomes. The LBWSAT and Sindh Cohort 1 studies reported intervention effects of approximately 0.15 and 0.26 WAZ scores, respectively (Saville et al., 2018; Soofi et al., 2022). However, the Cambodia CSB trial did not detect any significant differences in birthweight (Janmohamed, Karakochuk, Boungnasiri, Chapman, et al., 2016). Likewise, the Cambodia CSB trial did not detect any statistically significant differences in the prevalence of small‐for‐gestational‐age (Janmohamed, Karakochuk, Boungnasiri, Chapman, et al., 2016). The Sindh Cohort 1 study reported that birth length was 0.50 cm (*p* = 0.027) greater among infants whose mothers received WSB+ versus the SOC during pregnancy (Soofi et al., 2022). However, neither the Cambodia CSB study nor the LBWSAT study detected any difference in birth length between groups (Janmohamed, Karakochuk, Boungnasiri, Chapman, et al., 2016; Saville et al., 2018). The Sindh Cohort 1 study was the only trial that evaluated a measure of wasting at birth but did not detect any significant effects of WSB+ on this outcome (Soofi et al., 2022). Likewise, neither the Cambodia CSB nor the LBWSAT study detected any significant differences in birth head circumference between study groups (Janmohamed, Karakochuk, Boungnasiri, Chapman, et al., 2016; Saville et al., 2018). The Sindh Cohort 1 study was the only FBF trial that reported neonatal mortality as an outcome; however, significant differences were not detected (Soofi et al., 2022).

#### 3.3.3 Effects of multi‐component interventions that included food supplements on birth outcomes

The Oportunidades, PROCOMIDA, and WINGS trials assessed the effects of a multi‐component intervention that included a food supplement (fortified milk‐based beverage in Oportunidades; CSB or LNS in PROCOMIDA; IFA/MMS plus a snack and milk or egg in WINGS) on selected birth outcomes. Although it is not possible to isolate the effects of the food component alone, the Oportunidades trial detected a 127 g increase in birthweight among programme beneficiaries and a 4.6 pp reduction in low birthweight (Barber & Gertler, 2008). Similarly, the WINGS trial detected a 40 (8, 74) g and 78 (26, 129) g increase in birthweight in the preconception versus no preconception intervention group and comprehensive intervention versus SOC group, respectively (Taneja et al., 2022). These two comparisons also revealed significantly lower risks of low birthweight and small‐for‐gestational‐age (Taneja et al., 2022). The PROCOMIDA trial observed significantly greater LAZ scores among children at 1 month of age in the group that received the full family ration and CSB versus the control group but did not detect any significant differences in any of the other intervention groups (Olney et al., 2018). Similarly, the WINGS trial reported that birth length was 0.17 (0.01, 0.32) cm greater in the preconception versus no preconception intervention group but did not detect any significant effects of the other intervention groups (Taneja et al., 2022). Birth head circumference was also 0.12 (0.01, 0.23) cm greater in the pregnancy versus no pregnancy intervention group and 0.18 (0.02, 0.33) cm greater in the comprehensive intervention versus SOC group (Taneja et al., 2022). The WINGS trial was one of the few trials that was statistically powered to detect differences in neonatal mortality and reported a nearly 50% reduction in this outcome among the pregnancy (1.2%) versus no pregnancy (2.0%) intervention group (IRR: 0.52 [0.29, 0.95]) (Taneja et al., 2022).

### 3.4 Summary of infant/child outcomes

Data on infant/child growth and haemoglobin/anaemia were relatively sparse in relation to maternal and birth outcomes (Table 5, Supporting Information File 3: Table S6). There was also considerable variability in the age and frequency at which these outcomes were assessed. Infant outcomes were assessed in 7 trials at 3 months of age and in 14 trials at 6 months of age. Although infant outcomes beyond 6 months of age were frequently reported, the majority of trials (*n* = 11) provided supplemental fortified food (e.g., SQ‐LNS, CSB) to children starting at 6 months of age, which prevented the effects of maternal supplementation with fortified food during pregnancy and lactation from being isolated (Adu‐Afarwuah et al., 2015; Ashorn, Alho, Ashorn, Cheung, Dewey, Harjunmaa, et al., 2015; Galasso et al., 2019; Johnson et al., 2017; Leroy et al., 2008; Leroy et al., 2018; Matias et al., 2016; Neufeld et al., 2019; Olney et al., 2018; Soofi et al., 2022; Taneja et al., 2022); thus, these data were not extracted for the present review.

**Table 5:** Summary of effects on infant/child outcomes at approximately 3 and 6 months of age.†

Study name	Intervention groups	Child outcomes at ~ 3 months of age							Child outcomes at ~ 6 months of age						
		Weight/WAZ/underwt	Length/LAZ/stunting	WLZ/BMIZ/WLR/wasting	Head circ/HCAZ/low HCAZ	MUAC/low MUAC	Mortality	Hb/Anaemia	Weight/WAZ/underwt	Length/LAZ/stunting	WLZ/BMIZ/WLR/wasting	Head circ/HCAZ/low HCAZ	MUAC/low MUAC	Mortality	Hb/Anaemia
**LNS studies**															
Women First	1 = Arm 1 (pre‐conception SQ‐LNS); 2 = Arm 2 (SQ‐LNS at 12 weeks gestation); 3 = Control (SOC)														
iLiNS‐DYAD‐G‡	1 = SQ‐LNS; 2 = MMN; 3 = Control (IFA)														
iLiNS‐DYAD‐M‡	1 = SQ‐LNS; 2 = MMN; 3 = Control (IFA)														
RDNS	1 = SQ‐LNS‐PLW, SQ‐LNS‐C; 2 = IFA, SQ‐LNS‐C; 3 = IFA, MNP‐C; 4 = Control (IFA) [For outcomes, groups 2‐4 are combined as the control]														
Epi‐E	1 = 40g‐LNS; 2 = MMN; 3 = Control (IFA)														
MISAME‐II	1 = MQ‐LNS; 2 = Control (MMN)														
MISAME‐III (PRENATAL INTERVENTION)	1 = prenatal MQ‐LNS + IFA; 2 = postnatal MQ‐LNS + IFA; 3 = pre‐ and postnatal MQ‐LNS + IFA; 4 = Control (IFA) [For outcomes, groups 2 and 4 are combined as the control]														
MISAME‐III (POSTNATAL INTERVENTION)	1 = prenatal MQ‐LNS + IFA; 2 = postnatal MQ‐LNS + IFA; 3 = pre‐ and postnatal MQ‐LNS + IFA; 4 = Control (IFA) [For outcomes, groups 1 and 4 are combined as the control]														
ENID	1 = MMN; 2 = LQ‐LNS + IFA; 3 = LQ‐LNS + MMN; 4 = IFA														
BAN	1 = HAART; 2 = LQ‐LNS; 3 = HAART + LQ‐LNS; 4 = Control (SOC)														
**Supercereal (CSB+ or WSB** + **) studies**															
Sindh Cohort 1	1 = WSB + ; 2 = Control (SOC)														
Tubaramure	1 = T24 (CSB, pregnancy to 24 months), 2 = T18 (CSB, pregnancy to 18 mo), 3 = TNFP (CSB, birth to 24 months), 4 = Control (SOC)														
LBWSAT	1 = PLA only; 2 = PLA + Cash; 3 = PLA + WSB + ; 4 = Control														
**Other types of food supplements**															
Senegal	1 = Millet food supplement; 2 = Maize food supplement; 3 = Control (Non‐supplemented)														
IMPRINT	1 = Food Supplement (snack) + MMN; 2 = Control (SOC); both groups counselled on the availability of IFA, Ca and vitamin D through the national programme + BCC														
**Food supplement provided as part of a multi‐component intervention**															
PROCOMIDA	1 = FFR + CSB; 2 = RFR + CSB; 3 = NFR + CSB; 4 = FFR + SQ‐LNS; 5 = FFR + MNP; 6 = Control (SOC)														
WINGS	1 = Preconception and pregnancy and early childhood interventions; 2 = Preconception interventions only; 3 = Pregnancy and early childhood interventions only; 4 = Control (SOC)														

Abbreviations: BCC, behaviour change communication; BMIZ, body mass index‐for‐age *z*‐score; circ, circumference; circ, circumference; CSB, fortified corn–soy blend; CSB + , corn–soy blend fortified with updated vitamin and mineral premix; FFR, full family ration; HAART, highly active antiretroviral therapy; Hb, haemoglobin; HCAZ, head circumference‐for‐age *z*‐score; IFA, iron‐folic acid supplements; LAZ, length‐for‐age *z*‐score; LNS‐C, lipid‐based nutrient supplement for 6‐24 mo old children; LNS‐PBW, lipid‐based nutrient supplement for pregnant and breastfeeding women; LQ, large quantity; MMN, multiple micronutrients; MNP, micronutrient powder; MNP‐C, micronutrient powder for 6‐24 mo old children; MQ, medium quantity; MUAC, mid‐upper arm circumference; NFR, no family ration; PLA, participatory learning and action women's groups; RFR, reduced family ration; SOC, standard of care; SQ, small quantity; underwt, underweight; WAZ, weight‐for‐age *z*‐score; WLR, weight to length ratio; WLZ, weight‐for‐length *z*‐score; WSB + , wheat soy blend fortified with updated vitamin and mineral premix.

^†^ Green: positive/beneficial effect; grey: a null/nonsignificant effect on at least one of the related outcome variables; white, not measured or reported. *p* Values < 0.05 were considered statistically significant for the purposes of the synthesis activity. Outcomes are as defined by investigators. Detailed results are available in Supporting Information File 4: Table S6.

^‡^ Data from the iLiNS‐DYAD‐G and iLiNS‐DYAD‐M were provided by Charles Arnold, with permission from the trial investigators (Charles Arnold, personal communication, 15 December 2023).

#### 3.4.1 Effects of LNS on infant/child outcomes

Of the nine LNS trials, only the Women First, iLiNS‐DYAD‐G and Epi‐E trials reported measures of child growth at approximately 3 months of age. At this age, the Women First trial reported significant increases in weight of 102 g in the preconception SQ‐LNS versus control group and increases in length of 0.55 cm in the preconception SQ‐LNS group versus the control group and 0.4 cm increase in the group that initiated SQ‐LNS at 12 weeks gestation versus the SOC control but no differences in infant wasting or head circumference (Krebs et al., 2021). Similarly, the iLiNS‐DYAD‐G trial reported significant increases in weight and length of 149 g and 0.43 cm, respectively, in the SQ‐LNS versus IFA group, and a 0.21 SD greater WAZ in the SQ‐LNS versus MMN group, but no differences in wasting, head circumference or MUAC (Charles Arnold, personal communication, 15 December 2023). The Epi‐E trial did not detect any significant effects of MQ‐LNS on anthropometric outcomes assessed at 3 months of age (Bliznashka et al., 2022).

Few LNS trials reported any significant effects of the perinatal intervention on infant outcomes at 6 months of age. Exceptions to this included beneficial effects on weight/WAZ/underweight and length/LAZ/stunting that were demonstrated in the Women First and MISAME‐III trials. Although five LNS trials evaluated head circumference (Argaw et al., 2023; Dewey et al., 2017; Krebs et al., 2021), three evaluated MUAC (Argaw et al., 2023), one evaluated mortality (Krebs et al., 2021), and five evaluated anaemia/haemoglobin at 6 months of age (Adu‐Afarwuah et al., 2019; Argaw et al., 2023; Matias et al., 2018; Moore et al., 2019), none of these trials detected any significant differences in these outcomes among groups.

The Epi‐E, MISAME‐II, and MISAME‐III trials were the only LNS studies that evaluated at least one infant/child outcome above 6 months of age without providing LNS directly to children (Argaw et al., 2023; Bliznashka et al., 2022; Lanou et al., 2014). The Epi‐E and MISAME‐II trials did not detect any significant differences in measures of weight, underweight or wasting (Bliznashka et al., 2022; Lanou et al., 2014). The Epi‐E trial did not detect any significant differences in length at 24 months (Bliznashka et al., 2022), and the MISAME‐II and MISAME‐III trials reported mixed effects on child length. The only other significant anthropometric finding to note was from the Epi‐E trial, which revealed positive differences in MUAC of 0.4 and 0.5 cm among children at 24 months of age whose mothers received MQ‐LNS versus IFA and MQ‐LNS versus MMS, respectively (Bliznashka et al., 2022).

#### 3.4.2 Effects of FBF on infant/child outcomes

None of the three FBF trials measured any infant growth outcomes at 3 months of age. At 6 months of age, the Sindh Cohort 1 trial reported that the prevalences of underweight and stunting were respectively 11.2 and 12.5 percentage points lower among infants whose mothers received WSB+ versus the control group (Soofi et al., 2022). The Tubaramure and Sindh Cohort 1 studies evaluated measures of child wasting at 6 months of age; however, neither detected statistically significant differences between groups (Leroy et al., 2021; Soofi et al., 2022). The Tubaramure study was the only trial to assess haemoglobin/anaemia. Authors of this study reported a lower prevalence of anaemia among infants 0‐6 months of age in all intervention groups versus the control group (*p* < 0.05), and between 3 and 6 months of age a 0.1 g/dL increase in mean haemoglobin concentration among infants in the T24 versus the control group (*p* < 0.05) (Leroy et al., 2016). The LBWSAT trial was the only FBF trial that measured any infant outcomes above 6 months of age in non‐supplemented children but did not detect any significant differences in WAZ, LAZ, WLZ, or head circumference among 0–16 month old children (average age was 9 months) (Saville et al., 2018).

#### 3.4.3 Effects of multi‐component interventions that included food supplements on infant/child outcomes

The only infant outcome reported by the PROCOMIDA trial was infant length at 3 months and 6 months of age; however, no significant differences were detected between groups at either time point (Olney et al., 2018). The WINGS trial assessed multiple anthropometric measures at 6 months of age and consistently reported positive intervention effects of the multi‐component intervention (Taneja et al., 2022). WAZ, LAZ, and WLZ were all significantly greater in the pregnancy versus no pregnancy intervention group and in the comprehensive intervention versus SOC group, with effect sizes typically ranging between 0.10 and 0.22 *z*‐scores (Taneja et al., 2022). Similarly, head circumference and MUAC were both significantly greater in the pregnancy versus no pregnancy intervention group and in the comprehensive intervention versus SOC group, with effect sizes equalling 0.2 cm for head circumference and 0.2 cm for MUAC (Taneja et al., 2022).

### 3.5 Summary of acceptability and utilization

Nine trials conducted acceptability studies (7 LNS, 1 CSB + , 1 fortified milk‐based beverage), typically either before, or as a sub‐study in parallel with, a randomized controlled trial (Supporting Information File 4: Table S7) (Adu‐Afarwuah et al., 2011; Ali et al., 2015; Clermont et al., 2018; Harding et al., 2017; Janmohamed, Karakochuk, Boungnasiri, Whitfield, et al., 2016; Jones et al., 2021; Klevor et al., 2016; Lama, Khatry, et al., 2022; Lama, Moore, et al., 2022; Young et al., 2010). In seven of these studies, LNS and CSB+ had high overall acceptability (i.e., favourable organoleptic attributes, perceived health benefits, etc.). Common barriers to appropriate consumption included an aversion to the odour and taste during early pregnancy, concerns about delivering a large baby, and household food‐sharing practices. In one study, participants found the fortified milk‐based beverage acceptable but preferred MMS and MNP (Young et al., 2010). In only one acceptability study (MSF‐Bangladesh), the majority of participants found LNS unacceptable; in this study, participants were malnourished pregnant and lactating women receiving RUTF (LQ‐LNS) (Ali et al., 2015).

Ten of the randomized controlled trials provided information on LNS compliance/adherence/utilization among PBW/G during the intervention period. The majority of these studies (8 trials) were “efficacy trials”, in which all activities were conducted by the research teams, and the average observed or reported compliance (e.g., % of days LNS consumed, % women consumed LNS in the previous 24 h) was high (typically ≥ 75%) (Adu‐Afarwuah et al., 2016; Argaw et al., 2023; Ashorn, Alho, Ashorn, Cheung, Dewey, Gondwe, et al., 2015; Bliznashka et al., 2022; Flax et al., 2012; Hambidge et al., 2019; Huybregts et al., 2009; Moore et al., 2019) (Supporting Information File 4: Table S4). Two studies distributed LNS within existing community‐based programmes; one programme (MAHAY) reported a high reach (80% of enrolled women received LNS) (Galasso et al., 2019), the other (RDNS) reported 64% of participants were “high adherers” (consumed LNS ≥ 4 d/wk during pregnancy) (Mridha et al., 2016).

Four cluster‐randomized trials provided FBF (e.g., CSB + , WSB + ); the majority evaluated programmes integrated into the primary health care system or implemented by nongovernmental organizations. The average reported adherence (e.g., % of rations consumed, % women consumed FBF in the previous 24 h) was approximately 40%–60% (Janmohamed, Karakochuk, Boungnasiri, Chapman, et al., 2016; Leroy et al., 2018; Soofi et al., 2022). One programme reported a high reach (79% of enrolled women had received ≥4 monthly food rations) (Saville et al., 2018).

One additional study provided adherence data for both LNS and FBF, provided in different intervention arms. In Guatemala (PROCOMIDA), adherence (% of women consumed within the past 24 h at 4–6 mo post‐partum) to SQ‐LNS appeared higher (~60%) compared to CSB+ (~20%–40%) (Olney et al., 2018). Of note, consumption of CSB+ was greater (% ever consumed and % of women having consumed CSB+ within the past 24 h) among women receiving full family rations, compared to those receiving no family rations (Olney et al., 2018).

### 3.6 Review of reviews

Twenty‐eight review articles published within the previous 10 years were identified through PubMed and cross‐referencing (Supporting Information File 4: Table S8). Of these, 15 reported on systematic reviews (Bhutta et al., 2014; Das et al., 2018; Hofmeyr et al., 2023; Hunter et al., 2023; Keats, Oh, et al., 2021; Lassi et al., 2020, 2021; Oh et al., 2020; Ota et al., 2015; Park, Fang, et al., 2019; Park, Harari, et al., 2019; Park, Siden, et al., 2019; Ramakrishnan et al., 2014; Shah et al., 2021; Stevens et al., 2015), seven were narrative reviews (Adu‐Afarwuah, 2020; Adu‐Afarwuah, Lartey, & Dewey, 2017; Ciulei et al., 2023; Imdad & Bhutta, 2013; Mason et al., 2014; Sethi et al., 2021; Vaivada et al., 2017), five included reviews of reviews (Ciulei et al., 2023; Heidkamp et al., 2017; Ota et al., 2020; Visser et al., 2018; von Salmuth et al., 2021), one was a scoping review (Kurian et al., 2021) and one was a 2‐stage meta‐analysis of individual participant data (Liu et al., 2022). A brief summary of these reviews is provided in the online Supporting Information File 1: Results section 3.

### 3.7 Ongoing research

We identified 13 studies (9 LNS, 2 FBF, 2 other food supplements) that, upon their completion, may provide additional information on the outcomes of interest included in this review (Supporting Information File 4: Table S9). These trials address the efficacy/effectiveness of universal fortified BEP supplementation during pregnancy and/or lactation on maternal and infant outcomes, as well as the impact of targeting based on pre‐pregnancy BMI, inclusion of enhanced infection management, and effect of bundled interventions (e.g., family food rations, cash transfers in addition to supplemental fortified foods, etc.).

## 4 DISCUSSION

The present scoping review summarizes the available evidence on perinatal interventions providing food along with at least 3 micronutrients to PBW/G in LMICs. The intervention strategies of the 21 identified trials and programme evaluations with impact assessments were heterogeneous and included one or a combination of the following products along with behaviour change communication and other interventions: fortified LNS (*n* = 12), FBF (*n* = 5), milk‐based beverages (*n* = 2), and local food/snacks (*n* = 3) providing 118 kcal to over 750 kcal per day along with various levels of protein and micronutrients. Similarly, the evaluation designs and outcome assessments varied greatly. Effects on maternal outcomes such as gestational weight gain and gestational age at delivery were promising but inconsistent. Birth outcomes were reported more frequently, and the effects on foetal growth (i.e., birthweight and birth length) were generally positive, with some beneficial longer‐term impacts on infant and child growth in a few trials.

In conducting the scoping review, we were struck by the lack of clarity and consistency in how BEP supplementation for PBW/G has been defined. This is reflected in the broad range of energy, protein, and micronutrient composition of the supplements evaluated in different trials. Various numbers and amounts of micronutrients were added to the daily dose of LNS. Although the perinatal SQ‐LNS provided relatively low levels of energy (118 kcal) and protein (5.2 g) along with 22 micronutrients, they were specifically developed to complement the diet to meet the nutritional requirements during pregnancy and lactation (Arimond et al., 2015), and all four trials of SQ‐LNS evaluated a product with the same nutrient specifications. However, a subsequent Expert Consultation suggested that, in the context of undernutrition among PBW/G in low‐income settings, if an intervention is to make a sizable contribution in energy and protein to the increased requirements during pregnancy, a target of 14–18 g protein and 250–500 kcal/d (11–29% of energy from protein) should be provided (Members of an Expert Consultation on Nutritious Food Supplements for Pregnant and Lactating women, 2019). This recommendation informed the current specifications for MQ‐LNS used by WFP, UNICEF, and other organizations (UNICEF; WFP), which indicate that approximately 75 g of product per day should provide 14.1–16.7 g protein and 382–443 kcal/day. The quantity of LNS evaluated in the MISAME‐II and ‐III trials (de Kok et al., 2022; Huybregts et al., 2009), and the forthcoming MINT trial (Erchick et al., 2023), is consistent with these specifications. The nutrient composition of the fortified milk‐based beverage evaluated in the Prospera and Opportunidades trials also aligned with the recommendation of the Expert Consultation (Neufeld et al., 2019). However, of note, the energy content of the snack provided in the IMPRINT trial (Taneja et al., 2021) and LQ‐LNS provided in the ENID and BAN trials (Flax et al., 2012; Moore et al., 2012) was greater than 500 kcal. While four of the five FBF trials also provided a daily ration that exceeded 500 kcal/day, in many cases, the FBF was likely shared with other family members, and the woman's consumption would have been considerably less (Janmohamed, Karakochuk, Boungnasiri, Chapman, et al., 2016; Leroy et al., 2018; Saville et al., 2018; Soofi et al., 2022).

In our comprehensive assessment of the literature on LNS, we observed no notable or consistent variations in effects relative to the dosage or quantity of LNS provided. Although there was some evidence to indicate that LNS was effective in improving maternal nutritional status, the results were inconsistent. Two out of four trials found positive effects of SQ‐LNS on gestational weight gain, which is consistent with the results of a recent meta‐analysis of individual participant data that found no associations between SQ‐LNS and the percentage adequacy of gestational weight gain (Liu et al., 2022). While SQ‐LNS studies did not detect positive effects on gestational age at delivery, two trials of MQ‐LNS demonstrated such benefits (de Kok et al., 2022; Mohammad et al., 2022). In contrast, multiple trials of both SQ‐ and MQ‐LNS found beneficial effects on several birth outcomes, including birthweight/WAZ, birth length, head circumference, MUAC, and SGA. Similarly, a Cochrane analysis by Das et al. (2018) and a forthcoming meta‐analysis of individual participant data (Adu‐Afarwuah et al., 2023, 16–20 October; Wessells, Dewey, et al., 2021) found that antenatal SQ‐LNS and MMS had similar positive effects on birthweight outcomes when comparing each of these interventions with IFA. Considerably less evidence was available on the effects of perinatal LNS provision on infant/child growth; however, the Women First, iLiNS‐DYAD‐G, and MISAME‐III trials found some evidence of prolonged benefits of perinatal SQ‐LNS/MQ‐LNS on infant weight and length (Argaw et al., 2023; Krebs et al., 2021).

Although FBFs are widely used in programmes (WFP, 2023), we identified relatively few impact assessments of this class of products when provided to PBW/G. The Cambodia CSB trial identified consistently positive effects of CSB+ on the prevalence of inadequate gestational weight gain, preterm birth, and maternal anaemia but did not detect any significant differences in any birth outcomes that were measured (Janmohamed, Karakochuk, Boungnasiri, Chapman, et al., 2016). In contrast, the LBWSAT trial did not detect any differences in gestational age at delivery or preterm birth but did identify positive effects of WSB+ on birthweight and WAZ (Saville et al., 2018). The Sindh Cohort 1 study similarly identified positive effects of WSB+ on measures of both birthweight and length (Soofi et al., 2022).

Extensive assessments were conducted on the impact of maternal and child supplementation within the Progresa–Oportunidades–Prospera programme initiated by the Mexican government. However, we only found two articles explicitly addressing maternal and birth outcomes within the Oportunidades programme (Barber & Gertler, 2008; Leroy et al., 2008). The provision of a combination of cash and fortified foods during pregnancy and lactation had a positive impact on birthweight and the growth of infants <6 months of age, suggesting that the provision of cash and fortified foods to PBW/G had a positive impact on the offspring. A multi‐component intervention consisting of integrated and concurrent delivery of health, nutrition, water, sanitation and hygiene, and psychosocial care interventions (WINGS) (Taneja et al., 2022) demonstrated several positive impacts on maternal and infant outcomes, including gestational weight gain, birthweight, length and head circumference, and sustained anthropometric gains at 6 months of age. While the impact of the food supplements could not be isolated, bundled interventions are a promising approach to improve health and nutrition outcomes via multiple pathways as they can also address underlying causes of malnutrition.

The effects of the different interventions on maternal and child haemoglobin concentrations and/or anaemia deserve special comment, given the heterogeneity in these results. While most studies found no difference in anaemia prevalence between the LNS group compared with the IFA group, the iLiNS‐DYAD‐G and iLiNS‐DYAD‐M trials reported a higher prevalence of anaemia in the SQ‐LNS group (Adu‐Afarwuah, Lartey, Okronipa, Ashorn, Zeilani, et al., 2017; Jorgensen et al., 2018). It is important to note, however, that the dose of iron provided by SQ‐LNS and MMS was 20 mg versus 60 mg provided by the IFA supplement. The ENID trial, in which the LQ‐LNS provided the same amount of iron as the IFA supplement, also reported a lower haemoglobin concentration and higher prevalence of anaemia in the LQ‐LNS groups, which may have been related to lower‐than‐anticipated intakes of LQ‐LNS (Jobarteh et al., 2017). In contrast, in studies such as the Cambodia CSB trial, which detected a positive effect on anaemia, participants in the CSB+ group would have received approximately 13 mg of iron per day as well as other micronutrients (e.g. vitamin A, vitamin B12) that could improve haemoglobin if they consumed the full daily ration, whereas those in the SOC comparison would not have explicitly received any additional iron or other micronutrients (Janmohamed, Karakochuk, Boungnasiri, Chapman, et al., 2016). Interestingly, a recent analysis comparing MMS and IFA found that the MMS, including 30 mg of iron, had a similar impact as IFA, providing 60 mg of iron, on haemoglobin concentration, and risks of anaemia and iron deficiency anaemia (Gomes et al., 2022). Additional research and analysis are needed to better understand issues related to the iron dose of LNS and of other fortified BEPs as per the guidance of the Expert Consultation and the effects on other biomarkers of iron status. This is particularly important in view of the urgent need to accelerate action to prevent and manage anaemia (WHO, 2023).

The present scoping review is unique and differs from previous scoping and systematic reviews because our inclusion criteria required that any food or cash intervention strategy also provided three or more micronutrients either as part of the food product or as MMS. We adopted this approach given the strong evidence in support of MMS during pregnancy and lactation (Smith et al., 2017) and the recommendation by WHO to provide BEP to undernourished pregnant women to improve pregnancy outcomes (WHO, 2016).

We identified 177 eligible articles, which reported on 21 intervention trials or programme evaluations and 2 other types of trials, of which the majority were acceptability studies. The terminology used to describe the interventions ranged widely, requiring multiple modifications to our search terms. Thus, it is possible that we may have missed reports of other eligible intervention trials despite extensive cross‐checks of references. The heterogeneity in interventions, comparison groups, timing of initiation and duration of the intervention, and variety of outcome definitions make it difficult to draw firm conclusions. It is also important to note that over half of the published articles come from just 4 studies, all of which used SQ‐LNS, and that the evidence of MQ‐LNS and FBF comes from only 3 trials and 5 trials, respectively. However, current efforts to harmonize data for completed and ongoing maternal BEP studies will help to standardize variable definitions and outcome assessment to enhance consistency across protocols and enable comparisons to be drawn across trials (Gernand et al., 2023).

The present scoping review further underscores the need for additional research. Specifically, considering the positive impacts found in some LNS trials, the optimal dose and formulation of LNS needs to be determined in dose–response studies. Relatedly, more robust and systematic measures of adherence as well as net change of dietary intake are required. Data on the cost‐effectiveness of various doses of LNS, as well as different fortified BEP and local food/snack options, are also needed for policy and programme decisions. Several of the trials providing SQ‐LNS to children also provided perinatal SQ‐LNS to their mothers and found benefits on children's health outcomes (Dewey et al., 2022; Dewey, Wessells, et al., 2021; Prado et al., 2021; Stewart et al., 2020; Wessells, Arnold, et al., 2021). However, studies that combine maternal interventions during pregnancy and/or lactation with supplementation of the offspring after 6 months of age do not allow the long‐term impacts of maternal perinatal interventions on infant and young children's growth and other health outcomes to be isolated. Thus, the present evidence on the impacts of maternal perinatal interventions later in childhood is scarce. Research is needed to identify the optimal period and targeting approach for supplementation during the first 1000 days to maximize nutrition and health benefits. Finally, there is a need for further evidence on multi‐component interventions (i.e. bundled interventions that provide food with micronutrients along with other types of interventions) and interventions providing both cash or non‐fortified foods together with MMS to PBW/G. The provision of cash may increase the ability of PBW/G to meet their protein and energy intake and may also increase dietary diversity, but the provision of MMS would still be necessary to meet the high micronutrient requirements during this vulnerable period.

In summary, the evidence on the provision of BEP along with MMS, through fortification or as supplements, to PBW/G in LMICs on maternal outcomes and longer‐term infant and child growth is promising yet inconsistent. The evidence of beneficial impacts on birth outcomes, especially foetal growth, supports the role that these products can play in the global call for action to prevent childhood wasting (FAO, UNHCR, UNICEF, WFP, & WHO, 2023; UNICEF, FAO, UNHCR, WFP, & WHO, 2021). Further research is needed on the cost‐effectiveness of these products, particularly in programmatic settings, and to assess the impact of multi‐component interventions.

## AUTHOR CONTRIBUTIONS

Christine M. McDonald, K. Ryan Wessells, and Sonja Y. Hess performed the research and wrote the paper. Christine P. Stewart, Kathryn G. Dewey, Saskia de Pee, Ritu Rana, Hajra Hafeez‐ur‐Rehman and Martin N. Mwangi provided comments. All authors have read and approved the final manuscript.

## CONFLICT OF INTEREST STATEMENT

The authors declare no conflict of interest.

## Supporting information

Supporting information.

Supporting information.

Supporting information.

Supporting information.

## Data Availability

Data sharing does not apply to this article as no data sets were generated or analysed during the current study.
