# Supplementary material for: Perinatal intervention strategies providing food with micronutrients to pregnant and breastfeeding women in low‐ and middle‐income countries: A scoping review
Source: Matern Child Nutr. 2024 Jul 1;20(4):e13681. doi: 10.1111/mcn.13681 (PMC11574661; doi:10.1111/mcn.13681)
Supplement: Supplementary file 1 — Supporting information. [file MCN-20-e13681-s004.docx]

**Supplementary online results section**

**Perinatal intervention strategies providing food with micronutrients to pregnant and breastfeeding women in low- and middle-income countries: A scoping review**

Christine M. McDonald, K. Ryan Wessells, Christine P. Stewart, Kathryn G. Dewey, Saskia de Pee, Ritu Rana, Hajra Hafeez-ur-Rehman, Martin N. Mwangi, Sonja Y. Hess

Page

**Supplementary results 1.** Detailed overview of included studies 2

**Supplementary results 2.** Comparison of multiple types of food supplements 5

**Supplementary results 3.** Review of reviews 9

**Supplementary results section 1:** Detailed overview of included studies

Twenty-one trials assessed the nutrition or health impact of an eligible intervention vs. a control, and thus met the pre-defined eligibility criteria (**Figure 1** in main manuscript). Four trials each published 10-28 articles and contributed 54% of the articles overall (**Figure 2** in main manuscript). Over half of the studies (n=12) assessed the efficacy or effectiveness of LNS. Of these, five trials provided 20 g LNS/day, which is considered small-quantity (SQ-LNS) (Adu-Afarwuah, Lartey, Okronipa, Ashorn, Ashorn, et al., 2017; Ashorn, Alho, Ashorn, Cheung, Dewey, Harjunmaa, et al., 2015; Hambidge et al., 2019; Matias et al., 2016; Olney et al., 2018). Three trials provided 72-75 g LNS/day, which is considered medium-quantity (MQ-LNS) (de Kok et al., 2022; Huybregts et al., 2009; Mohammad et al., 2022), and two trials provided 140 g LNS/day, which is considered large-quantity (LQ-LNS) (Flax et al., 2012; Johnson et al., 2017). An additional two trials provided 40 g LNS/day (Galasso et al., 2019; Isanaka et al., 2021). In the Women First trial, women with a low BMI or inadequate gestational weight gain received 55 g/d of unfortified LNS in addition to the daily SQ-LNS (Hambidge et al., 2019). Four of these studies provided LNS during pregnancy only (Huybregts et al., 2009; Isanaka et al., 2021; Johnson et al., 2017; Mohammad et al., 2022), five during pregnancy and lactation (Adu-Afarwuah, Lartey, Okronipa, Ashorn, Ashorn, et al., 2017; Ashorn, Alho, Ashorn, Cheung, Dewey, Harjunmaa, et al., 2015; Galasso et al., 2019; Matias et al., 2016; Olney et al., 2018), one during pregnancy and/or lactation (de Kok et al., 2022), and one during lactation only (Flax et al., 2012). The Women First Study, which was implemented in 4 countries in parallel (Democratic Republic of the Congo, Guatemala, Indian, and Pakistan) compared the impact of providing SQ-LNS starting during the pre-conception period vs. initiation at 12-14 weeks of gestation (Hambidge et al., 2019). In all LNS studies, the daily dose of LNS was fortified with micronutrients but the composition and amounts varied (**Supplementary Table 10**). Three cluster randomized trials (Janmohamed, Karakochuk, Boungnasiri, Chapman, et al., 2016; Saville et al., 2018; Soofi, Khan, et al., 2022) and two program evaluations (Leroy et al., 2018; Olney et al., 2018) assessed the impact of providing FBF such as fortified corn soy blend (CSB or CSB+) or fortified wheat soy blend (WSB+) during pregnancy (n=2) (Janmohamed, Karakochuk, Boungnasiri, Chapman, et al., 2016; Saville et al., 2018), pregnancy and lactation (n=2) (Olney et al., 2018; Soofi, Khan, et al., 2022), or pregnancy and/or lactation (Leroy et al., 2018). Additional studies provided other types of foods (Cisse et al., 2002; Neufeld et al., 2019; Taneja et al., 2021). For example, the IMPRINT study assessed the provision of snacks (such as choco energy bites, panjeeri, jeera crackers, nut mixtures, and biscuits) along with MMS and behavior change communication (BCC) among breastfeeding women (Taneja et al., 2021). Three trials assessed the provision of supplemental foods as part of a multi-component intervention strategy (Oportunidades in Mexico, PROCOMIDA in Guatemala, and WINGS in India) (Leroy et al., 2008; Olney et al., 2018; Taneja et al., 2022).

Lastly, as described above, we also summarized five trials that did not include a control or non-intervention group, including the longitudinal INCAP study (maize-based atole vs. fresco, a caloric drink) (Delgado et al., 1982), a study by Ross et al. in which high-bulk vs. low-bulk food supplements were provided to pregnant women in South Africa (Ross et al., 1985), and MINIMat where the provision of supplemental foods was either started early (9 weeks gestation) or later in pregnancy (20 weeks gestation) as was usual in the study communities (Khan et al., 2011). Lastly, two of these latter studies assessed the impact of ready-to-use supplementary foods (RUSF) (LNS designed for treatment of moderate acute malnutrition) compared with CSB+ among malnourished pregnant women (Callaghan-Gillespie et al., 2017; Hendrixson et al., 2021). Although these five latter studies did not meet our pre-defined eligibility criteria, these studies provided important insights by assessing multiple types of food supplements and study findings are summarized in the online **Supplementary Results Section 2**.

Among all reviewed studies, SQ-LNS trials provided the lowest amount of supplemental food (118 kcal and 2.6 g protein/day) compared with LQ-LNS (746 kcal and 20.8 g protein/day) and FBF (600-700 kcal/day and 20-29 g protein/day from the cereal distributed with fortified oil, which provided an additional 90-180 kcal/day) (**Figure 3** in main manuscript). Moreover, some of these latter interventions provided an additional monthly ration of both FBF and oil for family sharing (Leroy et al., 2018; Saville et al., 2018). Distribution of the supplemental foods ranged from daily to monthly across all studies.

Although interventions providing cash along with micronutrient supplements were also of interest in the present scoping review, only the Oportunidades program implemented by the Mexican government included both cash transfers and fortified foods targeting PBW/G (Leroy et al., 2008). The LBWSAT study in Nepal included an intervention group that received cash (7.5 USD/mo). However, while all intervention groups benefited from participatory learning and action women's groups (PLA), the PLA + cash group did not receive micronutrient supplements, and the LBWSAT study was only eligible for inclusion in the present review because of the PLA + WSB+ intervention group (Saville et al., 2018).

**Supplementary results section 2:** Comparison of multiple types of food supplements

Here we also summarize five trials that did not include a control or non-intervention group. Although these studies did not meet our pre-defined eligible criteria, we determined that these studies provided important insights by assessing multiple types of food supplements such as the longitudinal INCAP study (maize-based atole vs. fresco, a caloric drink), a study by Ross et al. in which high-bulk vs. low-bulk food supplements were provided to pregnant women in South Africa, and MiniMat where the provision of supplemental foods was either started early (right after pregnancy identification) or later in pregnancy as usual in the study communities (**Table 2** in main manuscript). Lastly, two of these studies (Mamachiponde and Sierra Leone RUSF) assessed the impact of ready-to-use supplementary foods (RUSF) compared with CSB+ among malnourished pregnant women. While Mamachiponde provided RUSF in 250 g bottles, the study in Sierra Leone provided 100 g LNS.

*Comparison of multiple types of food supplements on maternal outcomes*

Of the three multi-product trials that evaluated gestational weight gain as an outcome, the Mamachiponde and Sierra Leone trials trial observed significantly greater weight gain among women who received RUSF vs. CSB+; however, the Ross trial did not detect any significant differences across intervention groups. The Sierra Leone RUSF trial also observed a greater increase in maternal MUAC among women who received RUSF vs. CSB+ plus IFA; however, the Mamachiponde trial did not detect any significant differences. In the INCAP and Sierra Leone RUSF trials, gestational age at birth was longer among women in the Atole vs. Fresco, and the RUSF vs. CSB+ plus IFA groups, respectively. However, neither the MiniMat nor the Ross trials detected any significant differences in gestational age or preterm birth. There were no significant differences in the incidence of stillbirth across groups among the three multi-product trials that evaluated this outcome. The MiniMat trial was the only study that reported measures of maternal anemia or iron status. This trial found that when micronutrient groups were combined, mean hemoglobin concentration was 0.9 g/dL lower among women in the early food supplementation group vs. late food supplementation group (p=0.04). However, there were no significant differences in iron status.

*Comparison of multiple types of food supplements on birth outcomes*

All five trials compared the effects of different types of food supplements on birth weight/WAZ. The INCAP and Sierra Leone RUSF trials observed increases in birth weight among the Atole vs. Fresco group and RUSF vs. SOC comparison group, respectively. The Mamachiponde and MiniMat trial did not detect any significant differences in this outcome between groups and the Ross trial reported significantly lower birth weights in the high-bulk, zinc supplement, and control group in comparison to the low-bulk supplement. Three trials evaluated low birth weight as an outcome. The Mamachiponde trial reported that the prevalence of low birth weight was 7 percentage points higher in the CSB + MMN group vs. IFA group; however, the MiniMat and Sierra Leone RUSF trials did not detect any differences in the outcomes between groups.

The Mamachiponde, MiniMat and Sierra Leone RUSF trials also assessed birth length/LAZ/newborn stunting, measures of wasting at birth, and birth head circumference as outcomes. All three trials did not detect any statistically significant differences in these outcomes with the exception of the Sierra Leone RUSF trial that identified a 0.3 (0.1, 0.6) cm increase in birth length in the intervention vs. control group. The Sierra Leone RUSF trial also detected a 0.1 (0.03, 0.02) cm increase in birth MUAC in the intervention vs. control group; however, the INCAP study did not detect any differences in this outcome between the Atole vs. Fresco group. Of note, the MiniMat and Sierra Leone RUSF trials reported significant effects of the intervention on neonatal mortality. The MiniMat trial reported a striking 58% reduction in neonatal mortality among the MMS + early food supplementation group vs. the 60 mg iron folic acid group (HR: 0.32 (0.13, 0.72) and the Sierra Leone RUSF trial reported that the neonatal mortality rate was 2.4 (0.30, 0.4) percentage points lower in the intervention vs. control group.

*Comparison of multiple types of food supplements on infant/child outcomes*

The Mamachiponde and MiniMat trials were the only two studies that evaluated any infant outcomes at 3 months of age. However, neither study detected significant differences in weight/WAZ or length/LAZ. The MiniMAT trial measured WLZ at 3 months, but did not detect any differences in this outcome between groups. Similarly, the Mamchiponde trial evaluated head circumference and MUAC at 3 months of age, but did not detect any significant differences in either outcome between groups.

At six months of age, the MiniMat trial did not detect any differences in WAZ, LAZ, or WLZ between groups. However, the Sierra Leone RUSF trial calculated an intervention estimate from linear mixed modeling of 0.09 (0.02, 0.15; p=0.01) kg in weight, 0.3 (0.1, 0.5; p=0.0114) in length, and 0.1 cm (0.01, 0.2; p=0.0398) at 6 months of age. No significant differences in head circumference were detected at six months of age. However, the mortality rate was 3.3 (0.2, 6.4) percentage points lower in the intervention vs. control group and the authors noted that the mortality benefit occurred in the first 21 days of life.

The MiniMat trial was the only study of different food supplements to evaluate child outcomes beyond six months of age. Although there were no significant differences in WAZ, HAZ, or WLZ at 54 months of age, the prevalence of stunting was 4.5 percentage points lower in the early vs. usual initiation groups (p=0.01). The MiniMat trial also reported a remarkable 66% reduction in under 5 mortality in the MMS + early food initiation vs. control group (HR = 0.32 (0.18, 0.65)).

**Supplementary results section 3:** Review of reviews

Twenty-eight review articles, published within the previous 10 years, were identified through PubMed and cross-referencing (**Supplementary table 8**). Of these, 15 reported on systematic reviews (Bhutta 2014, Ramakrishnan 2014, Ota 2015, Stevens 2015, Das 2018, Park 2019a, Park 2019b, Park 2019c, Lassi 2020, Oh 2020, Keats 2021, Lassi 2021, Shah 2021, Hofmeyr 2023, Hunter 2023), 7 were narrative reviews (Imdad 2013, Mason 2014, Adu-Afarwuah 2017, Vaivada 2017, Adu-Afarwuah 2020, Sethi 2021, Ciulei 2023), 5 included a review of reviews (Heidkamp 2017, Visser 2018, Otah 2020, von Salmuth 2021, Ciulei 2023), 1 was a scoping review (Kurian et al, 2021) and 1 was a 2-stage meta-analysis of individual participant data (Liu et al, 2022). The majority of these reviews focused on LMICs, 1 focused on conflict settings within LMIC (Shah 2021), while 3 took a regional focus on South Asia (Sethi 2021), on Southeast Asia and Pakistan (Kurian 2021) and on Sub-Saharan Africa (Adu-Afarwuah 2020). Most authors relied on the definition of pregnancy provided by the primary studies and did not specify other characteristics. However, there were two exceptions; the review of systematic reviews by Visser et al (2018) focused on food insecure, vulnerable and malnourished pregnant and lactating women, and the systematic review by Stevens et al. (2015) limited their meta-analysis to studies among women with undernutrition or only included study sub-groups of undernourished women.

The range of interventions included in the identified reviews varied widely, with 7 reviews considering a broad spectrum of nutrition and non-nutrition interventions that may affect the respective maternal, birth and/or infant outcome(s) of interest. An additional 12 reviews considered various prenatal, antenatal or postnatal nutrition interventions targeting either women or women and children. All of these reviews included BEP and/or LNS as one of the interventions of interest. Six reviews focused on the impact of LNS and three on the impact of BEP, and one review was interested in community-based supplementary feeding programs. Very few of the reviews provided a definition of BEP, and those that did used a definition of protein <25% of the total energy; most other authors relied on the definition provided by the primary studies. Importantly, none of the identified reviews required that BEP supplements were either fortified with ≥3 micronutrients or that micronutrient supplements were provided along with the food supplement. Nevertheless, there was a consistent conclusion among the included reviews that BEP supplementation with or without micronutrients had a positive impact on stillbirth and fetal growth (i.e., birthweight and SGA). Similarly, most reviews of LNS concluded that LNS had a positive impact on fetal growth (i.e., birthweight, length at birth, SGA) and gestational weight gain in some settings. In contrast, Keats et al. (2021) concluded that LNS made no difference on maternal, fetal and child health and development outcomes. Fewer reviews focused on supplementary food distribution. Visser et al (2018) concluded that the evidence of an impact among vulnerable populations was too limited and the evidence did not include outcomes such as quality of life and costs of programs.
