# Supplementary material for: Perinatal intervention strategies providing food with micronutrients to pregnant and breastfeeding women in low‐ and middle‐income countries: A scoping review
Source: Matern Child Nutr. 2024 Jul 1;20(4):e13681. doi: 10.1111/mcn.13681 (PMC11574661; doi:10.1111/mcn.13681)
Supplement: Supplementary file 2 — Supporting information. [file MCN-20-e13681-s001.docx]

**Supplementary online tables 1 – 3**

**Perinatal intervention strategies providing food with micronutrients to pregnant and breastfeeding women in low- and middle-income countries: A scoping review**

Christine M. McDonald, K. Ryan Wessells, Christine P. Stewart, Kathryn G. Dewey, Saskia de Pee, Ritu Rana, Hajra Hafeez-ur-Rehman, Martin N. Mwangi, Sonja Y. Hess

Page

**Supplementary table 1.** Pre-defined search terms for PubMed search 2

**Supplementary table 2.** Pre-defined search terms for trial registries 6

**Supplementary Table 3.** References by study reporting on the impact on 7
 health outcomes and/or acceptability

**Supplementary Table 1.** Pre-defined search terms for PubMed search

| **A** | **B** | **C** | **D** | **E** |
| --- | --- | --- | --- | --- |
| **Fortified products**  **(title/abstract)** | **Unfortified food/cash**  **(title/abstract)** | **Micronutrient  (all fields)** | **Population (title/abstract)** | **Location** |
| lipid near/3 supplement | "balanced energy protein" | micronutrient* | pregnan* | Extensive MeSH terms list for low-income and middle-income countries^†^ |
| lipid near/3 supplements | "balanced energy-protein" | vitamin* | prenatal |  |
| lipid near/3 nutrient | "balanced protein energy" | mineral* | pre-natal |  |
| lipid near/3 nutrients | "balanced protein-energy" | fortif* | perinatal |  |
| lipid near/3 fortified | "protein supplement*" | iron* | peri-natal |  |
| lipid near/3 fortificant | "energy supplement*" |  | antenatal |  |
| lipid near/3 fortificants | “food supplement*” |  | ante-natal |  |
| lipid near/3 enriched | "food assistance" |  | lactating |  |
| lipid near/3 powder | "food distribution" |  | breastfeeding |  |
| lipid near/3 powders | "food* specialized" |  | maternal |  |
| lipid near/3 spread | "food aid" |  |  |  |
| lipid near/3 spreads | "food ration*" |  |  |  |
| lipid near/3 paste | "food basket*" |  |  |  |
| Nutributter* | cash |  |  |  |
| Plumpy* | voucher* |  |  |  |
| "fortified blended food*" | coupon* |  |  |  |
| "specialized nutritious food*" | "supplement* food*" |  |  |  |
| "ready to use food*" | "supplement* feeding" |  |  |  |
| "corn soy blend*" | "nutrition assistance" |  |  |  |
| "wheat soy blend*" |  |  |  |  |
| “corn soya blend” |  |  |  |  |
| “wheat soya blend” |  |  |  |  |
| super cereal |  |  |  |  |
| supercereal |  |  |  |  |
| "high energy biscuit*" |  |  |  |  |
| "MAMTA" |  |  |  |  |
| "MUMTA" |  |  |  |  |
| "MAAMTA" |  |  |  |  |

^†^ From Ramafikeng et al (2022): ("emerging country"[all fields] OR "emerging countries"[all fields] OR "emerging nation"[all fields] OR "emerging nations"[all fields] OR "emerging population"[all fields] OR "emerging populations"[all fields] OR "developing country"[tiab] OR "developing countries"[tiab] OR "developing nation"[tiab] OR "developing nations"[tiab] OR "developing population"[tiab] OR "developing populations"[tiab] OR "developing world"[tiab] OR "less developed country"[tiab] OR "less developed countries"[tiab] OR "less developed nation"[tiab] OR "less developed nations"[tiab] OR "less developed population"[tiab] OR "less developed populations"[tiab] OR "less developed world"[tiab] OR "lesser developed country"[tiab] OR "lesser developed countries"[tiab] OR "lesser developed nation"[tiab] OR "lesser developed nations"[tiab] OR "lesser developed population"[tiab] OR "lesser developed populations"[tiab] OR "lesser developed world"[tiab] OR "under developed country"[tiab] OR "under developed countries"[tiab] OR "under developed nation"[tiab] OR "under developed nations"[tiab] OR "under developed population"[tiab] OR "under developed populations"[tiab] OR "under developed world"[tiab] OR "underdeveloped country"[tiab] OR "underdeveloped countries"[tiab] OR "underdeveloped nation"[tiab] OR "underdeveloped nations"[tiab] OR "underdeveloped population"[tiab] OR "underdeveloped populations"[tiab] OR "underdeveloped world"[tiab] OR "middle income country"[tiab] OR "middle income countries"[tiab] OR "middle income nation"[tiab] OR "middle income nations"[tiab] OR "middle income population"[tiab] OR "middle income populations"[tiab] OR "low income country"[tiab] OR "low income countries"[tiab] OR "low income nation"[tiab] OR "low income nations"[tiab] OR "low income population"[tiab] OR "low income populations"[tiab] OR "lower income country"[tiab] OR "lower income countries"[tiab] OR "lower income nation"[tiab] OR "lower income nations"[tiab] OR "lower income population"[tiab] OR "lower income populations"[tiab] OR "underserved country"[tiab] OR "underserved countries"[tiab] OR "underserved nation"[tiab] OR "underserved nations"[tiab] OR "underserved population"[tiab] OR "underserved populations"[tiab] OR "underserved world"[tiab] OR "under served country"[tiab] OR "under served countries"[tiab] OR "under served nation"[tiab] OR "under served nations"[tiab] OR "under served population"[tiab] OR "under served populations"[tiab] OR "under served world"[tiab] OR "deprived country"[tiab] OR "deprived countries"[tiab] OR "deprived nation"[tiab] OR "deprived nations"[tiab] OR "deprived population"[tiab] OR "deprived populations"[tiab] OR "deprived world"[tiab] OR "poor country"[tiab] OR "poor countries"[tiab] OR "poor nation"[tiab] OR "poor nations"[tiab] OR "poor population"[tiab] OR "poor populations"[tiab] OR "poor world"[tiab] OR "poorer country"[tiab] OR "poorer countries"[tiab] OR "poorer nation"[tiab] OR "poorer nations"[tiab] OR "poorer population"[tiab] OR "poorer populations"[tiab] OR "poorer world"[tiab] OR "developing economy"[tiab] OR "developing economies"[tiab] OR "less developed economy"[tiab] OR "less developed economies"[tiab] OR "lesser developed economy"[tiab] OR "lesser developed economies"[tiab] OR "under developed economy"[tiab] OR "under developed economies"[tiab] OR "underdeveloped economy"[tiab] OR "underdeveloped economies"[tiab] OR "middle income economy"[tiab] OR "middle income economies"[tiab] OR "low income economy"[tiab] OR "low income economies"[tiab] OR "lower income economy"[tiab] OR "lower income economies"[tiab] OR "low gdp"[tiab] OR "low gnp"[tiab] OR "low gross domestic"[tiab] OR "low gross national"[tiab] OR "lower gdp"[tiab] OR "lower gnp"[tiab] OR "lower gross domestic"[tiab] OR "lower gross national"[tiab] OR lmic[tiab] OR lmics[tiab] OR "third world"[tiab] OR "lami country"[tiab] OR "lami countries"[tiab] OR "transitional country"[tiab] OR "transitional countries"[tiab] OR Africa[tiab] OR Asia[tiab] OR Caribbean[tiab] OR West Indies[tiab] OR South America[tiab] OR Latin America[tiab] OR Central America[tiab] OR "Atlantic Islands"[tiab] OR "Commonwealth of Independent States"[tiab] OR "Pacific Islands"[tiab] OR "Indian Ocean Islands"[tiab] OR "Eastern Europe"[tiab] OR Afghanistan[tiab] OR Albania[tiab] OR Algeria[tiab] OR Angola[tiab] OR Antigua[tiab] OR Barbuda[tiab] OR Argentina[tiab] OR Armenia[tiab] OR Armenian[tiab] OR Aruba[tiab] OR Azerbaijan[tiab] OR Bahrain[tiab] OR Bangladesh[tiab] OR Barbados[tiab] OR Benin[tiab] OR Byelarus[tiab] OR Byelorussian[tiab] OR Belarus[tiab] OR Belorussian[tiab] OR Belorussia[tiab] OR Belize[tiab] OR Bhutan[tiab] OR Bolivia[tiab] OR Bosnia[tiab] OR Herzegovina[tiab] OR Hercegovina[tiab] OR Botswana[tiab] OR Brasil[tiab] OR Brazil[tiab] OR Bulgaria[tiab] OR Burkina Faso[tiab] OR Burkina Fasso[tiab] OR Upper Volta[tiab] OR Burundi[tiab] OR Urundi[tiab] OR Cambodia[tiab] OR Khmer Republic[tiab] OR Kampuchea[tiab] OR Cameroon[tiab] OR Cameroons[tiab] OR Cameron[tiab] OR Cape Verde[tiab] OR Central African Republic[tiab] OR Chad[tiab] OR Chile[tiab] OR China[tiab] OR Colombia[tiab] OR Comoros[tiab] OR Comoro Islands[tiab] OR Comores[tiab] OR Mayotte[tiab] OR Congo[tiab] OR Zaire[tiab] OR Costa Rica[tiab] OR Cote d'Ivoire[tiab] OR Ivory Coast[tiab] OR Croatia[tiab] OR Cuba[tiab] OR Cyprus[tiab] OR Czechoslovakia[tiab] OR "Czech Republic" [tiab] OR Slovakia[tiab] OR Slovak Republic[tiab] OR Djibouti[tiab] OR French Somaliland[tiab] OR Dominica[tiab] OR Dominican Republic[tiab] OR East Timor[tiab] OR East Timur[tiab] OR Timor Leste[tiab] OR Ecuador[tiab] OR Egypt[tiab] OR United Arab Republic[tiab] OR El Salvador[tiab] OR Eritrea[tiab] OR Estonia[tiab] OR Ethiopia[tiab] OR Fiji[tiab] OR Gabon[tiab] OR Gabonese Republic[tiab] OR Gambia[tiab] OR Gaza[tiab] OR Georgia Republic[tiab] OR Georgian Republic[tiab] OR Ghana[tiab] OR Gold Coast[tiab] OR Greece[tiab] OR Grenada[tiab] OR Guatemala[tiab] OR Guinea[tiab] OR Guam[tiab] OR Guiana[tiab] OR Guyana[tiab] OR Haiti[tiab] OR Honduras[tiab] OR Hungary[tiab] OR India[tiab] OR Maldives[tiab] OR Indonesia[tiab] OR Iran[tiab] OR Iraq[tiab] OR Jamaica[tiab] OR Jordan[tiab] OR Kazakhstan[tiab] OR Kazakh[tiab] OR Kenya[tiab] OR Kiribati[tiab] OR Korea[tiab] OR Kosovo[tiab] OR Kyrgyzstan[tiab] OR Kirghizia[tiab] OR Kyrgyz Republic[tiab] OR Kirghiz[tiab] OR Kirgizstan[tiab] OR "Lao PDR"[tiab] OR Laos[tiab] OR Latvia[tiab] OR Lebanon[tiab] OR Lesotho[tiab] OR Basutoland[tiab] OR Liberia[tiab] OR Libya[tiab] OR Lithuania[tiab]OR Macedonia[tiab] OR Madagascar[tiab] OR Malagasy Republic[tiab] OR Malaysia[tiab] OR Malaya[tiab] OR Malay[tiab] OR Sabah[tiab] OR Sarawak[tiab] OR Malawi[tiab] OR Nyasaland[tiab] OR Mali[tiab] OR Malta[tiab] OR Marshall Islands[tiab] OR Mauritania[tiab] OR Mauritius[tiab] OR Agalega Islands[tiab] OR "Melanesia"[tiab] OR Mexico[tiab] OR Micronesia[tiab] OR Middle East[tiab] OR Moldova[tiab] OR Moldovia[tiab] OR Moldovian[tiab] OR Mongolia[tiab] OR Montenegro[tiab] OR Morocco[tiab] OR Ifni[tiab] OR Mozambique[tiab] OR Myanmar[tiab] OR Myanma[tiab] OR Burma[tiab] OR Namibia[tiab] OR Nepal[tiab] OR Netherlands Antilles[tiab] OR New Caledonia[tiab] OR Nicaragua[tiab] OR Niger[tiab] OR Nigeria[tiab] OR Northern Mariana Islands[tiab] OR Oman[tiab] OR Muscat[tiab] OR Pakistan[tiab] OR Palau[tiab] OR Palestine[tiab] OR Panama[tiab] OR Paraguay[tiab] OR Peru[tiab] OR Philippines[tiab] OR Philipines[tiab] OR Phillipines[tiab] OR Phillippines[tiab] OR Poland[tiab] OR Portugal[tiab] OR Puerto Rico[tiab] OR Romania[tiab] OR Rumania[tiab] OR Roumania[tiab] OR Russia[tiab] OR Russian[tiab] OR Rwanda[tiab] OR Ruanda[tiab] OR Saint Kitts[tiab] OR St Kitts[tiab] OR Nevis[tiab] OR Saint Lucia[tiab] OR St Lucia[tiab] OR Saint Vincent[tiab] OR St Vincent[tiab] OR Grenadines[tiab] OR Samoa[tiab] OR Samoan Islands[tiab] OR Navigator Island[tiab] OR Navigator Islands[tiab] OR Sao Tome[tiab] OR Saudi Arabia[tiab] OR Senegal[tiab] OR Serbia[tiab] OR Montenegro[tiab] OR Seychelles[tiab] OR Sierra Leone[tiab] OR Slovenia[tiab] OR Sri Lanka[tiab] OR Ceylon[tiab] OR Solomon Islands[tiab] OR Somalia[tiab] OR Sudan[tiab] OR Suriname[tiab] OR Surinam[tiab] OR Swaziland[tiab] OR Syria[tiab] OR Syrian[tiab] OR Tajikistan[tiab] OR Tadzhikistan[tiab] OR Tadjikistan[tiab] OR Tadzhik[tiab] OR Tanzania[tiab] OR Thailand[tiab] OR Togo[tiab] OR Togolese Republic[tiab] OR Tonga[tiab] OR Trinidad[tiab] OR Tobago[tiab] OR Tunisia[tiab] OR Turkey[tiab] OR Turkmenistan[tiab] OR Turkmen[tiab] OR Tuvalu[tiab] OR Uganda[tiab] OR Ukraine[tiab] OR Uruguay[tiab] OR USSR[tiab] OR Soviet Union[tiab] OR Union of Soviet Socialist Republics[tiab] OR Uzbekistan[tiab] OR Uzbek OR Vanuatu[tiab] OR New Hebrides[tiab] OR Venezuela[tiab] OR Vietnam[tiab] OR Viet Nam[tiab] OR West Bank[tiab] OR Yemen[tiab] OR Yugoslavia[tiab] OR Zambia[tiab] OR Zimbabwe[tiab] OR Rhodesia[tiab] OR Developing Countries[Mesh] OR Africa[Mesh:NoExp] OR Africa, Northern[Mesh:NoExp] OR Africa South of the Sahara[Mesh:NoExp] OR Africa, Central[Mesh:NoExp] OR Africa, Eastern[Mesh:NoExp] OR Africa, Southern[Mesh:NoExp] OR Africa, Western[Mesh:NoExp] OR Asia[Mesh:NoExp] OR Asia, Central[Mesh:NoExp] OR Asia, Southeastern[Mesh:NoExp] OR Asia, Western[Mesh:NoExp] OR Caribbean Region[Mesh:NoExp] OR West Indies[Mesh:NoExp] OR South America[Mesh:NoExp] OR Latin America[Mesh:NoExp] OR Central America[Mesh:NoExp] OR "Atlantic Islands"[Mesh:NoExp] OR "Commonwealth of Independent States"[Mesh:NoExp] OR "Pacific Islands"[Mesh:NoExp] OR "Indian Ocean Islands"[Mesh:NoExp] OR "Europe, Eastern"[Mesh:NoExp] OR Afghanistan[Mesh] OR Albania[Mesh] OR Algeria[Mesh] OR American Samoa[Mesh] OR Angola[Mesh] OR "Antigua and Barbuda"[Mesh] OR Argentina[Mesh] OR Armenia[Mesh] OR Azerbaijan[Mesh] OR Bahrain[Mesh] OR "Baltic States"[Mesh] OR Bangladesh[Mesh] OR Barbados[Mesh] OR Benin[Mesh] OR "Republic of Belarus"[Mesh] OR Belize[Mesh] OR Bhutan[Mesh] OR Bolivia[Mesh] OR Bosnia-Herzegovina[Mesh] OR Botswana[Mesh] OR Brazil[Mesh] OR Bulgaria[Mesh] OR Burkina Faso[Mesh] OR Burundi[Mesh] OR Cambodia[Mesh] OR Cameroon[Mesh] OR Cape Verde[Mesh] OR Central African Republic[Mesh] OR Chad[Mesh] OR Chile[Mesh] OR China[Mesh] OR Colombia[Mesh] OR Comoros[Mesh] OR Congo[Mesh] OR Costa Rica[Mesh] OR Cote d'Ivoire[Mesh] OR Croatia[Mesh] OR Cuba[Mesh] OR Cyprus[Mesh] OR Czechoslovakia[Mesh] OR Czech Republic[Mesh] OR Slovakia[Mesh] OR Djibouti[Mesh] OR "Democratic Republic of the Congo"[Mesh] OR "Democratic People's Republic of Korea"[Mesh] OR Dominica[Mesh] OR Dominican Republic[Mesh] OR East Timor[Mesh] OR Ecuador[Mesh] OR Egypt[Mesh] OR El Salvador[Mesh] OR Eritrea[Mesh] OR Estonia[Mesh] OR Ethiopia[Mesh] OR "Equatorial Guinea"[Mesh] OR Fiji[Mesh] OR "French Guiana"[Mesh] OR Gabon[Mesh] OR Gambia[Mesh] OR "Georgia (Republic)"[Mesh] OR Ghana[Mesh] OR Greece[Mesh] OR Grenada[Mesh] OR Guatemala[Mesh] OR Guinea[Mesh] OR Guinea-Bissau[Mesh] OR Guam[Mesh] OR Guyana[Mesh] OR Haiti[Mesh] OR Honduras[Mesh] OR Hungary[Mesh] OR "Independent State of Samoa"[Mesh] OR India[Mesh] OR Indonesia[Mesh] OR Iran[Mesh] OR Iraq[Mesh] OR Jamaica[Mesh] OR Jordan[Mesh] OR Kazakhstan[Mesh] OR Kenya[Mesh] OR Korea[Mesh] OR Kyrgyzstan[Mesh] OR Laos[Mesh] OR Latvia[Mesh] OR Lebanon[Mesh] OR Lesotho[Mesh] OR Liberia[Mesh] OR Libya[Mesh] OR Lithuania[Mesh] OR "Macedonia (Republic)"[Mesh] OR Madagascar[Mesh] OR Malawi[Mesh] OR Malaysia[Mesh] OR Mali[Mesh] OR Malta[Mesh] OR Mauritania[Mesh] OR Mauritius[Mesh] OR "Melanesia"[Mesh] OR Mexico[Mesh] OR Micronesia[Mesh] OR Middle East[Mesh:NoExp] OR Moldova[Mesh] OR Mongolia[Mesh] OR Montenegro[Mesh] OR Morocco[Mesh] OR Mozambique[Mesh] OR Myanmar[Mesh] OR Namibia[Mesh] OR Nepal[Mesh] OR Netherlands Antilles[Mesh] OR New Caledonia[Mesh] OR Nicaragua[Mesh] OR Niger[Mesh] OR Nigeria[Mesh] OR Oman[Mesh] OR Pakistan[Mesh] OR Palau[Mesh] OR Panama[Mesh] OR Papua New Guinea[Mesh] OR Paraguay[Mesh] OR Peru[Mesh] OR Philippines[Mesh] OR Poland[Mesh] OR Portugal[Mesh] OR Puerto Rico[Mesh] OR "Republic of Korea"[Mesh] OR Romania[Mesh] OR Russia[Mesh] OR "Russia (Pre- 1917)"[Mesh] OR Rwanda[Mesh] OR "Saint Kitts and Nevis"[Mesh] OR Saint Lucia[Mesh] OR "Saint Vincent and the Grenadines"[Mesh] OR Samoa[Mesh] OR Saudi Arabia[Mesh] OR Senegal[Mesh] OR Serbia[Mesh] OR Montenegro[Mesh] OR Seychelles[Mesh] OR Sierra Leone[Mesh] OR Slovenia[Mesh] OR Sri Lanka[Mesh] OR Somalia[Mesh] OR South Africa[Mesh] OR Sudan[Mesh] OR Suriname[Mesh] OR Swaziland[Mesh] OR Syria[Mesh] OR Tajikistan[Mesh] OR Tanzania[Mesh] OR Thailand[Mesh] OR Togo[Mesh] OR Tonga[Mesh] OR "Trinidad and Tobago"[Mesh] OR Tunisia[Mesh] OR Turkey[Mesh] OR Turkmenistan[Mesh] OR Uganda[Mesh] OR Ukraine[Mesh] OR Uruguay[Mesh] OR USSR[Mesh] OR Uzbekistan[Mesh] OR Vanuatu[Mesh] OR Venezuela[Mesh] OR Vietnam[Mesh] OR Yemen[Mesh] OR Yugoslavia[Mesh] OR Zambia[Mesh] OR Zimbabwe[Mesh] OR "Southern African Development Community"[all fields] OR "East African Community"[all fields] OR "West African Health Organisation"[all fields] OR "Sub Saharan Africa "[all fields] OR "SubSaharan Africa "[all fields])

**Supplementary table 2.** Pre-defined search terms for trial registries

| **CLINICALTRIALS.GOV** |
| --- |
| **Date of search: October-11-2023** |
| (lipid supplement OR lipid supplements OR lipid nutrient OR lipid nutrients OR lipid fortified OR lipid fortificant OR lipid fortificants OR lipid enriched OR lipid powder OR lipid powders OR lipid spread OR lipid spreads OR lipid paste OR nutributter OR plumpy OR fortified blended food OR specialized nutritious food OR ready to use food OR corn soy blend OR corn soya blend OR wheat soya blend OR super cereal OR high energy biscuit OR MAMTA OR mumta OR ((balanced energy-protein OR balanced energy-protein OR balanced protein-energy OR balanced protein-energy OR protein supplement OR energy supplement OR food assistance OR food distribution OR food specialized OR food aid OR food ration OR food supplement OR food basket OR cash OR voucher OR coupon OR supplement food OR supplement feeding OR nutrition assistance) AND (micronutrient OR vitamin OR mineral OR fortif OR iron))) AND (pregnan OR prenatal OR pre-natal OR perinatal OR peri-natal OR antenatal OR ante-natal OR lactating OR breastfeeding OR maternal) |
| Eligibility criteria:   - Female - Adult (18-64 yrs) |
| Study status:   - Not yet recruiting - Recruiting - Active, not recruiting |
| No search term for location |
| **International Clinical Trials Registry Platform by the World Health Organization** |
| **Date of search: October-12-2023** |
| Intervention^†^:  (lipid supplement OR lipid supplements OR lipid nutrient OR lipid nutrients OR lipid fortified OR lipid fortificant OR lipid fortificants OR lipid enriched OR lipid powder OR lipid powders OR lipid spread OR lipid spreads OR lipid paste OR nutributter OR plumpy OR fortified blended food OR specialized nutritious food OR ready to use food OR corn soy blend OR corn soya blend OR wheat soya blend OR super cereal OR high energy biscuit OR MAMTA OR mumta OR ((balanced energy-protein OR balanced energy-protein OR balanced protein-energy OR balanced protein-energy OR protein supplement OR energy supplement OR food assistance OR food distribution OR food specialized OR food aid OR food ration OR food supplement OR food basket OR cash OR voucher OR coupon OR supplement food OR supplement feeding OR nutrition assistance) AND (micronutrient OR vitamin OR mineral OR fortif OR iron))) |
| All recruitment status |
| Date of registration between 01/01/2018 and 10/12/2023 |
| No search term for location |

^†^ Search was restricted to 1000 characters, so did not include the following terms: AND (pregnan OR prenatal OR pre-natal OR perinatal OR peri-natal OR antenatal OR ante-natal OR lactating OR breastfeeding OR maternal)

**Supplementary Table 3**. References by study reporting the impact on maternal, birth and/or child outcomes and/or acceptability ^†^

| **Study name** | **Publications** | **Primary reference** |
| --- | --- | --- |
| **LNS studies** | | |
| Women First | Borengasser 2018 (1), Castillo-Castrejon 2021 (2), Dhaded 2020 (3), Fernandes 2023 (4), Gilley 2020 (5), Hambidge 2019 (6), Krebs 2021 (7), Tang 2022 (8), Young 2021 (9), Young 2021 (10) | Hambidge 2019 (6) |
| iLiNS-DYAD-G^‡^ | Abreu 2021 (11), Adams 2018 (12), Adams 2020 (13), Adu-Afarwuah 2011(14), Adu-Afarwuah 2015 (15), Adu-Afarwuah 2016 (16), Adu-Afarwuah 2017 (17), Adu-Afarwuah 2017 (18), Adu-Afarwuah 2018 (19), Adu-Afarwuah 2018 (20), Adu-Afarwuah 2019 (21), Adu-Afarwuah 2020 (22), Adu-Afarwuah 2022 (23), Adu-Afarwuah 2023 (24), Haskell 2021 (25), Hong 2021 (26), Klevor 2016 (27), Klevor 2016 (28), Kumordzie 2019 (29), Kumordzie 2019 (30), Oaks 2016 (31), Oaks 2017 (32), Oaks 2020 (33), Ocansey 2019 (34), Ocansey 2019 (35), Okronipa 2018 (36), Prado 2016 (37), Prado 2023 (38) | Adu-Afarwuah 2017 (17) |
| iLiNS-DYAD-M^‡^ | Ashorn 2015 (39), Ashorn 2015 (40), Adu-Afarwuah 2021 (41), Adu-Afarwuah 2022 (23), Adu-Afarwuah 2023 (24), Barua 2018 (42), Chandrasiri 2015 (43), Harjunmaa 2016 (44), Haskell 2021 (25), Haskell 2022 (45), Jorgensen 2017 (46), Jorgensen 2018 (47), Kamng'ona 2020 (48), Klevor 2016 (27), Oaks 2017 (32), Prado 2016 (49), Prado 2018 (50), Pulakka 2017 (51), Salenius 2022 (52), Stewart 2015 (53), Stewart 2017 (54) | Ashorn 2015 (40) |
| RDNS | Dewey 2017 (55), Harding 2017 (56), Matias 2016 (57), Matias 2017 (58), Matias 2018 (59), Matias 2018 (60), Mridha 2016 (61), Mridha 2017 (62), Mridha 2017 (63), Ullah 2019 (64), Ullah 2019 (65) | Matias 2016 (57) |
| Epi-E | Bliznashka 2022 (66), Clermont 2018 (67), Isanaka 2019 (68), Isanaka 2021 (69), Sudfeld 2022 (70) | Isanaka 2021 (69) |
| MAHAY | Galasso 2019 (71), Stewart 2020 (72) | Galasso 2019 (71) |
| MINT | Lama 2022 (73), Lama 2022 (74) | Lama 2022 (73) |
| MISAME-II | Huybregts 2009 (75), Huybregts 2013 (76), Lanou 2014 (77), Toe 2015 (78) | Huybregts 2009 (75) |
| MISAME-III | Argaw 2023 (79), Argaw 2023 (80), Argaw 2023 (81), de Kok 2021 (82), de Kok 2021 (83), de Kok 2022 (84), Hanley-Cook 2022 (85), Hanley-Cook 2022 (86), Jones 2021 (87) | de Kok 2022 (84) |
| Pakistan Pre-E | Mohammad 2022 (88), Sher 2022 (89) | Mohammad 2022 (88) |
| ENID | Jobarteh 2017 (90), Johnson 2017 (91), Jones 2020 (92), Moore 2019 (93) | Johnson 2017 (91) |
| MSF-Bangladesh | Ali 2015 (94) | Ali 2015 (94) |
| BAN | Allen 2015 (95), Flax 2012 (96), Flax 2014 (97), Flax 2015 (98), Hampel 2018 (99), Kayira 2012 (100), Widen 2015 (101) | Flax 2012 (96) |
| **Super cereal (CSB+ or WSB+) studies** | | |
| Cambodia CSB | Janmohamed 2016 (102), Janmohamed 2016 (103) | Janmohamed 2016 (102) |
| Sindh Cohort 1 | Khan 2021 (104), Soofi 2022 (105), Zaidi 2020 (106) | Soofi 2022 (105) |
| Tubaramure | Leroy 2016 (107), Leroy 2018 (108), Leroy 2020 (109), Leroy 2021 (110), Olney 2019 (111) | Leroy 2018 (108) |
| LBWSAT | Harris-Fry 2018 (112), Saville 2018 (113) | Saville 2018 (113) |
| **Other types of food supplements** | | |
| IMPRINT | Taneja 2021 (114) | Taneja 2021 (114) |
| Prospera^¶^ | Mejia-Rodriguez 2022 (115), Neufeld 2019 (116), Young 2010 (117) | Neufeld 2019 (116) |
| Senegal | Cisse 2002 (118) | Cisse 2002 (118) |
| **Food supplement provided as part of a bundled intervention** | | |
| Oportunidades^¶^ | Barber 2008 (119), Leroy 2008 (120) | Leroy 2008 (120) |
| PROCOMIDA | Leroy 2019 (121), Olney 2018 (122) | Olney 2018 (122) |
| WINGS | Taneja 2022 (123) | Taneja 2022 (123) |
| **Studies comparing multiple types of food supplements** | | |
| INCAP^§^ | Barrett 1985 (124), Delgado 1982 (125), Delgado 1982 (126), Lechtig 1975 (127), Martorell 2020 (128), Webb 2005 (129) | Delgado 1982 (125) |
| MINIMat | Ekstrom 2016 (130), Eneroth 2010 (131), Hawkesworth 2013 (132), Islam Khan 2013 (133), Kallioinen 2017 (134), Khan 2011 (135), Khan 2015 (136), Khan 2017 (137), Persson 2012 (138), Shaheen 2014 (139), Shaheen 2015 (140), Siddiqua 2022 (141), Svefors 2018 (142), Tofail 2008 (143), Ziaei 2016 (144) | Khan 2011 (135) |
| South Africa Ross | Ross 1985 (145) | Ross 1985 (145) |
| Mamachiponde | Callaghan-Gillespie 2017 (146), Glosz 2018 (147) | Callaghan-Gillespie 2017 (146) |
| Sierra Leone RUSF | Hendrixson 2021 (148), Koroma 2023 (149) | Hendrixson 2021 (148) |

^†^ Manuscript describing study protocols were not considered eligible for the scoping review, but were consulted for the description of the study design when needed.
^‡^ Five articles reported on outcomes collected in two trials. Data for 3- and 6-month infant outcomes were provided by Charles Arnold, with permission from the trial investigators (Charles Arnold, personal communication, December 15, 2023). ^§^ Several additional articles reported re-analyses of the INCAP study results

^¶^ The trials referred here as ‘Prospera’ and ‘Oportunidades’ were evaluations of different aspects of the ‘cash-transfer Progresa - Oportunidades – Prospera’ program implemented by the Mexican government. Several additional articles reported analyses of the program.

**References**

**Women First**

1. Borengasser S.J., Baker P.R., Kerns M.E., Miller L.V., Palacios A.P., Kemp J.F., . . . Krebs N.F. Preconception micronutrient supplementation reduced circulating branched chain amino acids at 12 weeks gestation in an open trial of Guatemalan women who are overweight or obese. Nutrients 2018;10:11.

2. Castillo-Castrejon M., Yang I.V., Davidson E.J., Borengasser S.J., Jambal P., Westcott J., . . . Powell T.L. Preconceptional lipid-based nutrient supplementation in 2 low-resource countries results in distinctly different IGF-1/mTOR placental responses. Journal of Nutrition 2021;151:556-69.

3. Dhaded S.M., Hambidge K.M., Ali S.A., Somannavar M., Saleem S., Pasha O., . . . Krebs N.F. Preconception nutrition intervention improved birth length and reduced stunting and wasting in newborns in South Asia: The Women First randomized controlled trial. PLoS ONE 2020;15:e0218960.

4. Fernandes M., Krebs N.F., Westcott J., Tshefu A., Lokangaka A., Bauserman M., . . . Women First Preconception Nutrition Trial Study Group. Neurodevelopment, vision and auditory outcomes at age 2 years in offspring of participants in the 'Women First' maternal preconception nutrition randomised controlled trial. Archives of Disease in Childhood 2023;108:622-31.

5. Gilley S.P., Weaver N.E., Sticca E.L., Jambal P., Palacios A., Kerns M.E., . . . Borengasser S.J. Longitudinal changes of one-carbon metabolites and amino acid concentrations during pregnancy in the Women First maternal nutrition trial. Current Developments in Nutrition 2020;4:nzz132.

6. Hambidge K.M., Westcott J.E., Garces A., Figueroa L., Goudar S.S., Dhaded S.M., . . . Women First Preconception Trial Study Group. A multicountry randomized controlled trial of comprehensive maternal nutrition supplementation initiated before conception: The Women First trial. American Journal of Clinical Nutrition 2019;109:457-69.

7. Krebs N.F., Hambidge K.M., Westcott J.L., Garces A.L., Figueroa L., Tsefu A.K., . . . Women First Preconception Maternal Nutrition Study Group. Growth from birth through six months for infants of mothers in the "Women First" preconception maternal nutrition trial. Journal of Pediatrics 2021;229:199-206.e4.

8. Tang M., Weaver N.E., Frank D.N., Ir D., Robertson C.E., Kemp J.F., . . . Krebs N.F. Longitudinal reduction in diversity of maternal gut microbiota during pregnancy is observed in multiple low-resource settings: Results from the Women First trial. Frontiers in Microbiology 2022;13:823757.

9. Young A.E., Kemp J.F., Uhlson C., Westcott J.L., Ali S.A., Saleem S., . . . Women First Preconception Maternal Nutrition Trial Group. Improved first trimester maternal iodine status with preconception supplementation: The Women First trial. Maternal & Child Nutrition 2021:e13204.

10. Young B.E., Westcott J., Kemp J., Allen L., Hampel D., Garces A.L., . . . Women First Working Group. B-vitamins and choline in human milk are not impacted by a preconception lipid-based nutrient supplement, but differ among three low-to-middle income settings-findings from the Women First trial. Frontiers in Nutrition 2021;8:750680.

**iLiNS-DYAD-G**

11. Abreu A.M., Young R.R., Buchanan A., Lofgren I.E., Okronipa H.E.T., Lartey A., . . . Oaks B.M. Maternal blood pressure in relation to prenatal lipid-based nutrient supplementation and adverse birth outcomes in a Ghanaian cohort: A randomized controlled trial and cohort analysis. Journal of Nutrition 2021;151:1637-45.

12. Adams K.P., Okronipa H., Adu-Afarwuah S., Arimond M., Kumordzie S., Oaks B.M., . . . Dewey K.G. Ghanaian parents' perceptions of pre and postnatal nutrient supplements and their effects. Maternal & Child Nutrition 2018;14:e12608.

13. Adams K.P., Adu-Afarwuah S., Mridha M.K., Oaks B.M., Matias S.L., Arnold C.D., . . . Dewey K.G. The impact of maternal supplementation during pregnancy and the first 6 months postpartum on the growth status of the next child born after the intervention period: Follow-up results from Bangladesh and Ghana. Maternal & Child Nutrition 2020;16:e12927.

14. Adu-Afarwuah S., Lartey A., Zeilani M., Dewey K.G. Acceptability of lipid-based nutrient supplements (LNS) among Ghanaian infants and pregnant or lactating women. Maternal & Child Nutrition 2011;7:344-56.

15. Adu-Afarwuah S., Lartey A., Okronipa H., Ashorn P., Zeilani M., Peerson J.M., . . . Dewey K.G. Lipid-based nutrient supplement increases the birth size of infants of primiparous women in Ghana. American Journal of Clinical Nutrition 2015;101:835-46.

16. Adu-Afarwuah S., Lartey A., Okronipa H., Ashorn P., Peerson J.M., Arimond M., . . . Dewey K.G. Small-quantity, lipid-based nutrient supplements provided to women during pregnancy and 6 mo postpartum and to their infants from 6 mo of age increase the mean attained length of 18-mo-old children in semi-urban Ghana: A randomized controlled trial. American Journal of Clinical Nutrition 2016;104:797-808.

17. Adu-Afarwuah S., Lartey A., Okronipa H., Ashorn P., Ashorn U., Zeilani M., . . . Dewey K.G. Maternal supplementation with small-quantity lipid-based nutrient supplements compared with multiple micronutrients, but not with iron and folic acid, reduces the prevalence of low gestational weight gain in semi-urban Ghana: A randomized controlled trial. Journal of Nutrition 2017;147:697-705.

18. Adu-Afarwuah S., Lartey A., Okronipa H., Ashorn P., Zeilani M., Baldiviez L.M., . . . Dewey K.G. Impact of small-quantity lipid-based nutrient supplement on hemoglobin, iron status and biomarkers of inflammation in pregnant Ghanaian women. Maternal & Child Nutrition 2017;13.

19. Adu-Afarwuah S. From the field: Improving fetal and infant growth in vulnerable populations. Food & Nutrition Bulletin 2018;39:S60-S8.

20. Adu-Afarwuah S., Young R.T., Lartey A., Okronipa H., Ashorn P., Ashorn U., . . . Dewey K.G. Supplementation during pregnancy with small-quantity lipid-based nutrient supplements or multiple micronutrients, compared with iron and folic acid, increases women's urinary iodine concentration in semiurban Ghana: A randomized controlled trial. Maternal & Child Nutrition 2018;14:e12570.

21. Adu-Afarwuah S., Young R.T., Lartey A., Okronipa H., Ashorn P., Ashorn U., . . . Dewey K.G. Maternal and infant supplementation with small-quantity lipid-based nutrient supplements increases infants' iron status at 18 months of age in a semiurban setting in Ghana: A secondary outcome analysis of the iLiNS-DYAD randomized controlled trial. Journal of Nutrition 2019;149:149-58.

22. Adu-Afarwuah S., Young R.R., Lartey A., Okronipa H., Ashorn P., Ashorn U., . . . Dewey K.G. Supplementation with small-quantity lipid-based nutrient supplements does not increase child morbidity in a semiurban setting in Ghana: A secondary outcome noninferiority analysis of the international lipid-based nutrient supplements (iLiNS)-DYAD randomized controlled trial. Journal of Nutrition 2020;150:382-93.

23. Adu-Afarwuah S., Arnold C.D., Lartey A., Okronipa H., Maleta K., Ashorn P., . . . Dewey K.G. Small-quantity lipid-based nutrient supplements increase infants' plasma essential fatty acid levels in Ghana and Malawi: A secondary outcome analysis of the iLiNS-DYAD randomized trials. Journal of Nutrition 2022;152:286-301.

24. Adu-Afarwuah S., Arnold C.D., Lartey A., Okronipa H., Maleta K., Ashorn P., . . . Dewey K.G. Prevalence of morbidity symptoms among pregnant and postpartum women receiving different nutrient supplements in Ghana and Malawi: A secondary outcome analysis of two randomised controlled trials. Maternal & Child Nutrition 2023;19:e13501.

25. Haskell M.J., Young R., Adu-Afaruwah S., Lartey A., Okronipa H.E.T., Maleta K., . . . Dewey K.G. Small-quantity lipid-based nutrient supplements do not affect plasma or milk retinol concentrations among Malawian mothers, or plasma retinol concentrations among young Malawian or Ghanaian children in two randomized trials. Journal of Nutrition 2021;151:1029-37.

26. Hong B.V., Zhu C., Wong M., Sacchi R., Rhodes C.H., Kang J.W., . . . Zivkovic A.M. Lipid-based nutrient supplementation increases high-density lipoprotein (HDL) cholesterol efflux capacity and is associated with changes in the HDL glycoproteome in children. ACS Omega 2021;6:32022-31.

27. Klevor M.K., Adu-Afarwuah S., Ashorn P., Arimond M., Dewey K.G., Lartey A., . . . Ashorn U. A mixed method study exploring adherence to and acceptability of small quantity lipid-based nutrient supplements (SQ-LNS) among pregnant and lactating women in Ghana and Malawi. BMC Pregnancy & Childbirth 2016;16:253.

28. Klevor M.K., Haskell M.J., Lartey A., Adu-Afarwuah S., Zeilani M., Dewey K.G. Lipid-based nutrient supplements providing approximately the recommended daily intake of vitamin a do not increase breast milk retinol concentrations among Ghanaian women. Journal of Nutrition 2016;146:335-42.

29. Kumordzie S.M., Adu-Afarwuah S., Arimond M., Young R.R., Adom T., Boatin R., . . . Dewey K.G. Maternal and infant lipid-based nutritional supplementation increases height of Ghanaian children at 4-6 years only if the mother was not overweight before conception. Journal of Nutrition 2019;149:847-55.

30. Kumordzie S.M., Adu-Afarwuah S., Young R.R., Oaks B.M., Tamakloe S.M., Ocansey M.E., . . . Dewey K.G. Maternal-infant supplementation with small-quantity lipid-based nutrient supplements does not affect child blood pressure at 4-6 y in Ghana: Follow-up of a randomized trial. Journal of Nutrition 2019;149:522-31.

31. Oaks B.M., Laugero K.D., Stewart C.P., Adu-Afarwuah S., Lartey A., Ashorn P., . . . Dewey K.G. Late-pregnancy salivary cortisol concentrations of Ghanaian women participating in a randomized controlled trial of prenatal lipid-based nutrient supplements. Journal of Nutrition 2016;146:343-52.

32. Oaks B.M., Young R.R., Adu-Afarwuah S., Ashorn U., Jackson K.H., Lartey A., . . . Dewey K.G. Effects of a lipid-based nutrient supplement during pregnancy and lactation on maternal plasma fatty acid status and lipid profile: Results of two randomized controlled trials. Prostaglandins, Leukotrienes and Essential Fatty Acids 2017;117:28-35.

33. Oaks B.M., Adu-Afarwuah S., Kumordzie S., Laudenslager M.L., Smith D.L., Lin J., . . . Dewey K.G. Impact of a nutritional supplement during gestation and early childhood on child salivary cortisol, hair cortisol, and telomere length at 4-6 years of age: A follow-up of a randomized controlled trial. Stress 2020:1-10.

34. Ocansey M.E., Adu-Afarwuah S., Kumordzie S.M., Okronipa H., Young R.R., Tamakloe S.M., . . . Prado E.L. Prenatal and postnatal lipid-based nutrient supplementation and cognitive, social-emotional, and motor function in preschool-aged children in Ghana: A follow-up of a randomized controlled trial. American Journal of Clinical Nutrition 2019;109:322-34.

35. Ocansey M.E., Pulakka A., Adu-Afarwuah S., Young R.R., Kumordzie S.M., Okronipa H., . . . Prado E.L. The effects of supplementing maternal and infant diets with lipid-based nutrient supplements on physical activity and sedentary behaviour at preschool age in Ghana. British Journal of Nutrition 2019;122:884-94.

36. Okronipa H., Adu-Afarwuah S., Lartey A., Ashorn P., Vosti S.A., Young R.R., Dewey K.G. Maternal supplementation with small-quantity lipid-based nutrient supplements during pregnancy and lactation does not reduce depressive symptoms at 6 months postpartum in Ghanaian women: A randomized controlled trial. Archives of Women’s Mental Health 2018;21:55-63.

37. Prado E.L., Adu-Afarwuah S., Lartey A., Ocansey M., Ashorn P., Vosti S.A., Dewey K.G. Effects of pre- and post-natal lipid-based nutrient supplements on infant development in a randomized trial in Ghana. Early Human Development 2016;99:43-51.

38. Prado E.L., Adu-Afarwuah S., Arnold C.D., Adjetey E., Amponsah B., Bentil H., . . . Hastings P.D. Prenatal and postnatal small-quantity lipid-based nutrient supplements and children's social-emotional difficulties at ages 9-11 y inGghana: Follow-up of a randomized controlled trial. American Journal of Clinical Nutrition 2023;118:433-42.

**iLiNS-DYAD-M**

39. Ashorn P., Alho L., Ashorn U., Cheung Y.B., Dewey K.G., Gondwe A., . . . Maleta K. Supplementation of maternal diets during pregnancy and for 6 months postpartum and infant diets thereafter with small-quantity lipid-based nutrient supplements does not promote child growth by 18 months of age in rural Malawi: A randomized controlled trial. Journal of Nutrition 2015;145:1345-53.

40. Ashorn P., Alho L., Ashorn U., Cheung Y.B., Dewey K.G., Harjunmaa U., . . . Maleta K. The impact of lipid-based nutrient supplement provision to pregnant women on newborn size in rural Malawi: A randomized controlled trial. American Journal of Clinical Nutrition 2015;101:387-97.

41. Adu-Afarwuah S., Arnold C.D., Maleta K., Ashorn P., Ashorn U., Jorgensen J.M., . . . Dewey K.G. Consumption of multiple micronutrients or small-quantity lipid-based nutrient supplements containing iodine at the recommended dose during pregnancy, compared with iron and folic acid, does not affect women's urinary iodine concentration in rural Malawi: A secondary outcome analysis of the iLiNS DYAD trial. Public Health Nutrition 2021;24:3049-57.

42. Barua P., Chandrasiri U.P., Beeson J.G., Dewey K.G., Maleta K., Ashorn P., Rogerson S.J. Effect of nutrient supplementation on the acquisition of humoral immunity to Plasmodium falciparum in young Malawian children. Malaria Journal 2018;17:74.

43. Chandrasiri U.P., Fowkes F.J., Richards J.S., Langer C., Fan Y.M., Taylor S.M., . . . Rogerson S.J. The impact of lipid-based nutrient supplementation on anti-malarial antibodies in pregnant women in a randomized controlled trial. Malaria Journal 2015;14:193.

44. Harjunmaa U., Jarnstedt J., Dewey K.G., Ashorn U., Maleta K., Vosti S.A., Ashorn P. Nutrient supplementation may adversely affect maternal oral health--a randomised controlled trial in rural Malawi. Maternal & Child Nutrition 2016;12:99-110.

45. Haskell M.J., Maleta K., Arnold C.D., Jorgensen J.M., Fan Y.M., Ashorn U., . . . Dewey K.G. Provision of small-quantity lipid-based nutrient supplements increases plasma selenium concentration in pregnant women in Malawi: A secondary outcome of a randomized controlled trial. Current Developments in Nutrition 2022;6:nzac013.

46. Jorgensen J.M., Arnold C., Ashorn P., Ashorn U., Chaima D., Cheung Y.B., . . . Dewey K.G. Lipid-based nutrient supplements during pregnancy and lactation did not affect human milk oligosaccharides and bioactive proteins in a randomized trial. Journal of Nutrition 2017;147:1867-74.

47. Jorgensen J.M., Ashorn P., Ashorn U., Baldiviez L.M., Gondwe A., Maleta K., . . . Dewey K.G. Effects of lipid-based nutrient supplements or multiple micronutrient supplements compared with iron and folic acid supplements during pregnancy on maternal haemoglobin and iron status. Maternal & Child Nutrition 2018;14:e12640.

48. Kamng'ona A.W., Young R., Arnold C.D., Patson N., Jorgensen J.M., Kortekangas E., . . . Dewey K.G. Provision of lipid-based nutrient supplements to mothers during pregnancy and 6 months postpartum and to their infants from 6 to 18 months promotes infant gut microbiota diversity at 18 months of age but not microbiota maturation in a rural Malawian setting: Secondary outcomes of a randomized trial. Journal of Nutrition 2020;150:918-28.

49. Prado E.L., Maleta K., Ashorn P., Ashorn U., Vosti S.A., Sadalaki J., Dewey K.G. Effects of maternal and child lipid-based nutrient supplements on infant development: A randomized trial in Malawi. American Journal of Clinical Nutrition 2016;103:784-93.

50. Prado E.L., Ashorn U., Phuka J., Maleta K., Sadalaki J., Oaks B.M., . . . Dewey K.G. Associations of maternal nutrition during pregnancy and post-partum with maternal cognition and caregiving. Maternal & Child Nutrition 2018;14:e12546.

51. Pulakka A., Cheung Y.B., Maleta K., Dewey K.G., Kumwenda C., Bendabenda J., . . . Ashorn P. Effect of 12-month intervention with lipid-based nutrient supplement on the physical activity of Malawian toddlers: A randomised, controlled trial. British Journal of Nutrition 2017;117:511-8.

52. Salenius M., Pyykkö J., Ashorn U., Dewey K.G., Gondwe A., Harjunmaa U., . . . Adubra L. Association between prenatal provision of lipid‐based nutrient supplements and caesarean delivery: Findings from a randomised controlled trial in Malawi. Maternal & Child Nutrition 2022;18:1-10.

53. Stewart C.P., Oaks B.M., Laugero K.D., Ashorn U., Harjunmaa U., Kumwenda C., . . . Dewey K.G. Maternal cortisol and stress are associated with birth outcomes, but are not affected by lipid-based nutrient supplements during pregnancy: An analysis of data from a randomized controlled trial in rural Malawi. BMC Pregnancy & Childbirth 2015;15:346.

54. Stewart R.C., Ashorn P., Umar E., Dewey K.G., Ashorn U., Creed F., . . . Maleta K. The impact of maternal diet fortification with lipid-based nutrient supplements on postpartum depression in rural Malawi: A randomised-controlled trial. Maternal & Child Nutrition 2017;13.

**RDNS**

55. Dewey K.G., Mridha M.K., Matias S.L., Arnold C.D., Cummins J.R., Khan M.S., . . . Vosti S.A. Lipid-based nutrient supplementation in the first 1000 d improves child growth in Bangladesh: A cluster-randomized effectiveness trial. American Journal of Clinical Nutrition 2017;105:944-57.

56. Harding K.L., Matias S.L., Mridha M.K., Moniruzzaman M., Vosti S.A., Hussain S., . . . Stewart C.P. Adherence to recommendations on lipid-based nutrient supplement and iron and folic acid tablet consumption among pregnant and lactating women participating in a community health programme in northwest Bangladesh. Maternal & Child Nutrition 2017;13.

57. Matias S.L., Mridha M.K., Paul R.R., Hussain S., Vosti S.A., Arnold C.D., Dewey K.G. Prenatal lipid-based nutrient supplements affect maternal anthropometric indicators only in certain subgroups of rural Bangladeshi women. Journal of Nutrition 2016;146:1775-82.

58. Matias S.L., Mridha M.K., Tofail F., Arnold C.D., Khan M.S., Siddiqui Z., . . . Dewey K.G. Home fortification during the first 1000 d improves child development in Bangladesh: A cluster-randomized effectiveness trial. American Journal of Clinical Nutrition 2017;105:958-69.

59. Matias S.L., Mridha M.K., Young R.T., Hussain S., Dewey K.G. Daily maternal lipid-based nutrient supplementation with 20 mg iron, compared with iron and folic acid with 60 mg iron, resulted in lower iron status in late pregnancy but not at 6 months postpartum in either the mothers or their infants in Bangladesh. Journal of Nutrition 2018;148:1615-24.

60. Matias S.L., Mridha M.K., Young R.T., Khan M.S.A., Siddiqui Z., Ullah M.B., . . . Dewey K.G. Prenatal and postnatal supplementation with lipid-based nutrient supplements reduces anemia and iron deficiency in 18-month-old Bangladeshi children: A cluster-randomized effectiveness trial. Journal of Nutrition 2018;148:1167-76.

61. Mridha M.K., Matias S.L., Chaparro C.M., Paul R.R., Hussain S., Vosti S.A., . . . Dewey K.G. Lipid-based nutrient supplements for pregnant women reduce newborn stunting in a cluster-randomized controlled effectiveness trial in Bangladesh. American Journal of Clinical Nutrition 2016;103:236-49.

62. Mridha M.K., Matias S.L., Paul R.R., Hussain S., Khan M.S.A., Siddiqui Z., . . . Dewey K.G. Daily consumption of lipid-based nutrient supplements containing 250 mcg iodine does not increase urinary iodine concentrations in pregnant and postpartum women in Bangladesh. Journal of Nutrition 2017;147:1586-92.

63. Mridha M.K., Matias S.L., Paul R.R., Hussain S., Sarker M., Hossain M., . . . Dewey K.G. Prenatal lipid-based nutrient supplements do not affect pregnancy or childbirth complications or cesarean delivery in Bangladesh: A cluster-randomized controlled effectiveness trial. Journal of Nutrition 2017;147:1776-84.

64. Ullah M.B., Mridha M.K., Arnold C.D., Matias S.L., Khan M.S.A., Siddiqui Z., . . . Dewey K.G. Provision of pre- and postnatal nutritional supplements generally did not increase or decrease common childhood illnesses in Bangladesh: A cluster-randomized effectiveness trial. Journal of Nutrition 2019;149:1271-81.

65. Ullah M.B., Mridha M.K., Arnold C.D., Matias S.L., Khan M.S.A., Siddiqui Z., . . . Dewey K.G. Newborn physical condition and breastfeeding behaviours: Secondary outcomes of a cluster-randomized trial of prenatal lipid-based nutrient supplements in Bangladesh. Maternal & Child Nutrition 2019;15:e12844.

**Epi-E**

66. Bliznashka L., Sudfeld C.R., Garba S., Guindo O., Soumana I., Adehossi I., . . . Isanaka S. Prenatal supplementation with multiple micronutrient supplements or medium-quantity lipid-based nutrient supplements has limited effects on child growth up to 24 months in rural Niger: A secondary analysis of a cluster randomized trial. American Journal of Clinical Nutrition 2022;115:738-48.

67. Clermont A., Kodish S.R., Matar Seck A., Salifou A., Rosen J., Grais R.F., Isanaka S. Acceptability and utilization of three nutritional supplements during pregnancy: Findings from a longitudinal, mixed-methods study in Niger. Nutrients 2018;10.

68. Isanaka S., Kodish S.R., Mamaty A.A., Guindo O., Zeilani M., Grais R.F. Acceptability and utilization of a lipid-based nutrient supplement formulated for pregnant women in rural Niger: A multi-methods study. BMC Nutrition 2019;5:34.

69. Isanaka S., Garba S., Plikaytis B., Malone McNeal M., Guindo O., Langendorf C., . . . Grais R.F. Immunogenicity of an oral rotavirus vaccine administered with prenatal nutritional support in Niger: A cluster randomized clinical trial. PLoS Medicine 2021;18:e1003720.

70. Sudfeld C.R., Bliznashka L., Salifou A., Guindo O., Soumana I., Adehossi I., . . . Isanaka S. Evaluation of multiple micronutrient supplementation and medium-quantity lipid-based nutrient supplementation in pregnancy on child development in rural Niger: A secondary analysis of a cluster randomized controlled trial. PLOS Medicine 2022;19:1-17.

**MAHAY**

71. Galasso E., Weber A.M., Stewart C.P., Ratsifandrihamanana L., Fernald L.C.H. Effects of nutritional supplementation and home visiting on growth and development in young children in Madagascar: A cluster-randomised controlled trial. Lancet Global Health 2019;7:e1257-e68.

72. Stewart C.P., Fernald L.C.H., Weber A.M., Arnold C., Galasso E. Lipid-based nutrient supplementation reduces child anemia and increases micronutrient status in Madagascar: A multiarm cluster-randomized controlled trial. Journal of Nutrition 2020;150:958-66.

**MINT**

73. Lama T.P., Khatry S.K., Isanaka S., Moore K., Jones L., Bedford J., . . . Tielsch J.M. Acceptability of 11 fortified balanced energy-protein supplements for pregnant women in Nepal. Maternal & Child Nutrition 2022;18:e13336.

74. Lama T.P., Moore K., Isanaka S., Jones L., Bedford J., de Pee S., . . . Tielsch J.M. Compliance with and acceptability of two fortified balanced energy protein supplements among pregnant women in rural Nepal. Maternal & Child Nutrition 2022;18:1-15.

**MISAME-II**

75. Huybregts L., Roberfroid D., Lanou H., Menten J., Meda N., Van Camp J., Kolsteren P. Prenatal food supplementation fortified with multiple micronutrients increases birth length: A randomized controlled trial in rural Burkina Faso. American Journal of Clinical Nutrition 2009;90:1593-600.

76. Huybregts L., Roberfroid D., Lanou H., Meda N., Taes Y., Valea I., . . . Van Camp J. Prenatal lipid-based nutrient supplements increase cord leptin concentration in pregnant women from rural Burkina Faso. Journal of Nutrition 2013;143:576-83.

77. Lanou H., Huybregts L., Roberfroid D., Nikiema L., Kouanda S., Van Camp J., Kolsteren P. Prenatal nutrient supplementation and postnatal growth in a developing nation: An RCT. Pediatrics 2014;133:e1001-8.

78. Toe L.C., Bouckaert K.P., De Beuf K., Roberfroid D., Meda N., Thas O., . . . Huybregts L.F. Seasonality modifies the effect of a lipid-based nutrient supplement for pregnant rural women on birth length. Journal of Nutrition 2015;145:634-9.

**MISAME-III**

79. Argaw A., de Kok B., Toe L.C., Hanley-Cook G., Dailey-Chwalibog T., Ouedraogo M., . . . Huybregts L. Fortified balanced energy-protein supplementation during pregnancy and lactation and infant growth in rural Burkina Faso: A 2 x 2 factorial individually randomized controlled trial. PLOS Medicine 2023;20:e1004186.

80. Argaw A., de Kok B., Toe L.C., Hanley-Cook G., Dailey-Chwalibog T., Ouedraogo M., . . . Huybregts L. Correction: Fortified balanced energy-protein supplementation during pregnancy and lactation and infant growth in rural Burkina Faso: A 2 x 2 factorial individually randomized controlled trial. PLoS Med 2023;20:e1004267.

81. Argaw A., Toe L.C., Hanley-Cook G., Dailey-Chwalibog T., de Kok B., Ouedraogo L., . . . Huybregts L. Effect of prenatal micronutrient-fortified balanced energy-protein supplementation on maternal and newborn body composition: A sub-study from the MISAME-III randomized controlled efficacy trial in rural Burkina Faso. PLoS Medicine 2023;20:e1004242.

82. de Kok B., Argaw A., Hanley-Cook G., Toe L.C., Ouedraogo M., Dailey-Chwalibog T., . . . Huybregts L. Fortified balanced energy-protein supplements increase nutrient adequacy without displacing food intake in pregnant women in rural Burkina Faso. Journal of Nutrition 2021;07:07.

83. de Kok B., Moore K., Jones L., Vanslambrouck K., Toe L.C., Ouedraogo M., . . . Isanaka S. Home consumption of two fortified balanced energy protein supplements by pregnant women in Burkina Faso. Maternal & Child Nutrition 2021;17:e13134.

84. de Kok B., Toe L.C., Hanley-Cook G., Argaw A., Ouedraogo M., Compaore A., . . . Lachat C. Prenatal fortified balanced energy-protein supplementation and birth outcomes in rural Burkina Faso: A randomized controlled efficacy trial. PLOS Medicine 2022;19.

85. Hanley-Cook G., Toe L.C., Tesfamariam K., de Kok B., Argaw A., Compaore A., . . . Huybregts L. Fortified balanced energy-protein supplementation, maternal anemia, and gestational weight gain: A randomized controlled efficacy trial among pregnant women in rural Burkina Faso. Journal of Nutrition 2022;152:2277-86.

86. Hanley-Cook G.T., Argaw A., de Kok B., Toe L.C., Dailey-Chwalibog T., Ouedraogo M., . . . Lachat C. Seasonality and day-to-day variability of dietary diversity: Longitudinal study of pregnant women enrolled in a randomized controlled efficacy trial in rural Burkina Faso. Journal of Nutrition 2022;152:2145-54.

87. Jones L., de Kok B., Moore K., de Pee S., Bedford J., Vanslambrouck K., . . . Isanaka S. Acceptability of 12 fortified balanced energy protein supplements - insights from Burkina Faso. Maternal & Child Nutrition 2021;17:e13067.

**Pakistan Pre-E**

88. Mohammad N.S., Nazli R., Zafar H., Fatima S. Effects of lipid based multiple micronutrients supplement on the birth outcome of underweight pre-eclamptic women: A randomized clinical trial. Pakistan Journal of Medical Sciences 2022;38:219-26.

89. Sher N., Mubaraki M.A., Zafar H., Nazli R., Zafar M., Fatima S., Fozia F. Effect of lipid-based multiple micronutrients supplementation in underweight primigravida pre-eclamptic women on maternal and pregnancy outcomes: Randomized clinical trial. Medicina (Kaunas) 2022;58.

**ENID**

90. Jobarteh M.L., McArdle H.J., Holtrop G., Sise E.A., Prentice A.M., Moore S.E. mRNA levels of placental iron and zinc transporter genes are upregulated in Gambian women with low iron and zinc status. Journal of Nutrition 2017;147:1401-9.

91. Johnson W., Darboe M.K., Sosseh F., Nshe P., Prentice A.M., Moore S.E. Association of prenatal lipid-based nutritional supplementation with fetal growth in rural Gambia. Maternal & Child Nutrition 2017;13.

92. Jones K.S., Meadows S.R., Schoenmakers I., Prentice A., Moore S.E. Vitamin D status increases during pregnancy and in response to vitamin D supplementation in rural Gambian women. Journal of Nutrition 2020;150:492-504.

93. Moore S.E., Fulford A.J.C., Sosseh F., Nshe P., Darboe M.K., Prentice A.M. Thymic size is increased by infancy, but not pregnancy, nutritional supplementation in rural Gambian children: A randomized clinical trial. BMC Medicine 2019;17:38.

**MSF-Bangladesh**

94. Ali E., Zachariah R., Shams Z., Manzi M., Akter T., Alders P., . . . Harries A.D. Peanut-based ready-to-use therapeutic food: How acceptable and tolerated is it among malnourished pregnant and lactating women in Bangladesh? Maternal & Child Nutrition 2015;11:1028-35.

**BAN**

95. Allen L.H., Hampel D., Shahab-Ferdows S., York E.R., Adair L.S., Flax V.L., . . . Bentley M.E. Antiretroviral therapy provided to HIV-infected Malawian women in a randomized trial diminishes the positive effects of lipid-based nutrient supplements on breast-milk B vitamins. American Journal of Clinical Nutrition 2015;102:1468-74.

96. Flax V.L., Bentley M.E., Chasela C.S., Kayira D., Hudgens M.G., Knight R.J., . . . Adair L.S. Use of lipid-based nutrient supplements by HIV-infected malawian women during lactation has no effect on infant growth from 0 to 24 weeks. Journal of Nutrition 2012;142:1350-6.

97. Flax V.L., Bentley M.E., Combs G.F., Jr., Chasela C.S., Kayira D., Tegha G., . . . Adair L.S. Plasma and breast-milk selenium in HIV-infected Malawian mothers are positively associated with infant selenium status but are not associated with maternal supplementation: Results of the breastfeeding, antiretrovirals, and nutrition study. American Journal of Clinical Nutrition 2014;99:950-6.

98. Flax V.L., Adair L.S., Allen L.H., Shahab-Ferdows S., Hampel D., Chasela C.S., . . . B. A. N. Study Team. Plasma micronutrient concentrations are altered by antiretroviral therapy and lipid-based nutrient supplements in lactating HIV-infected malawian women. Journal of Nutrition 2015;145:1950-7.

99. Hampel D., Shahab-Ferdows S., Gertz E., Flax V.L., Adair L.S., Bentley M.E., . . . Allen L.H. The effects of a lipid-based nutrient supplement and antiretroviral therapy in a randomized controlled trial on iron, copper, and zinc in milk from HIV-infected Malawian mothers and associations with maternal and infant biomarkers. Maternal & Child Nutrition 2018;14:e12503.

100. Kayira D., Bentley M.E., Wiener J., Mkhomawanthu C., King C.C., Chitsulo P., . . . B.A.N. Study Team. A lipid-based nutrient supplement mitigates weight loss among HIV-infected women in a factorial randomized trial to prevent mother-to-child transmission during exclusive breastfeeding. American Journal of Clinical Nutrition 2012;95:759-65.

101. Widen E.M., Bentley M.E., Chasela C.S., Kayira D., Flax V.L., Kourtis A.P., . . .B.A.N. Study Team. Antiretroviral treatment is associated with iron deficiency in HIV-infected malawian women that is mitigated with supplementation, but is not associated with infant iron deficiency during 24 weeks of exclusive breastfeeding. Journal of Acquired Immune Deficiency Syndrome 2015;69:319-28.

**Cambodia CSB**

102. Janmohamed A., Karakochuk C.D., Boungnasiri S., Chapman G.E., Janssen P.A., Brant R., . . . McLean J. Prenatal supplementation with Corn Soya Blend Plus reduces the risk of maternal anemia in late gestation and lowers the rate of preterm birth but does not significantly improve maternal weight gain and birth anthropometric measurements in rural Cambodian women: A randomized trial. American Journal of Clinical Nutrition 2016;103:559-66.

103. Janmohamed A., Karakochuk C.D., Boungnasiri S., Whitfield K.C., Chapman G.E., Janssen P., . . . Green T.J. Factors affecting the acceptability and consumption of Corn Soya Blend Plus as a prenatal dietary supplement among pregnant women in rural Cambodia. Public Health Nutrition 2016;19:1842-51.

**Sindh Cohort 1**

104. Khan G.N., Ariff S., Kureishy S., Sajid M., Rizvi A., Garzon C., . . . Bhutta Z.A. Effectiveness of Wheat Soya Blend supplementation during pregnancy and lactation on pregnancy outcomes and nutritional status of their infants at 6 months of age in Thatta and Sujawal districts of Sindh, Pakistan: A cluster randomized-controlled trial. European Journal of Nutrition 2021;60:781-9.

105. Soofi S., Khan G., Ariff S., Ihtesham Y., Tanimoune M., Rizvi A., . . . Bhutta Z. Effectiveness of nutritional supplementation during the first 1000-days of life to reduce child undernutrition: A cluster randomized controlled trial in Pakistan. The Lancet Regional Health - Southeast Asia 2022;4.

106. Zaidi S., Das J.K., Khan G.N., Najmi R., Shah M.M., Soofi S.B. Food supplements to reduce stunting in Pakistan: A process evaluation of community dynamics shaping uptake. BMC Public Health 2020;20:1046.

**Tubaramure**

107. Leroy J.L., Olney D., Ruel M. Tubaramure, a food-assisted integrated health and nutrition program in Burundi, increases maternal and child hemoglobin concentrations and reduces anemia: A theory-based cluster-randomized controlled intervention trial. Journal of Nutrition 2016;146:1601-8.

108. Leroy J.L., Olney D., Ruel M. Tubaramure, a food-assisted integrated health and nutrition program, reduces child stunting in Burundi: A cluster-randomized controlled intervention trial. Journal of Nutrition 2018;148:445-52.

109. Leroy J.L., D K.O., Bliznashka L., Ruel M. Tubaramure, a food-assisted maternal and child health and nutrition program in Burundi, increased household food security and energy and micronutrient consumption, and maternal and child dietary diversity: A cluster-randomized controlled trial. Journal of Nutrition 2020;150:945-57.

110. Leroy J.L., Olney D.K., Nduwabike N., Ruel M.T. Tubaramure, a food-assisted integrated health and nutrition program, reduces child wasting in Burundi: A cluster-randomized controlled intervention trial. Journal of Nutrition 2021;151:197-205.

111. Olney D.K., Leroy J.L., Bliznashka L., Ruel M.T. A multisectoral food-assisted maternal and child health and nutrition program targeted to women and children in the first 1000 days increases attainment of language and motor milestones among young Burundian children. Journal of Nutrition 2019;149:1833-42.

**LBWSAT**

112. Harris-Fry H.A., Paudel P., Harrisson T., Shrestha N., Jha S., Beard B.J., . . . Saville N.M. Participatory women's groups with cash transfers can increase dietary diversity and micronutrient adequacy during pregnancy, whereas women's groups with food transfers can increase equity in intrahousehold energy allocation. Journal of Nutrition 2018;148:1472-83.

113. Saville N.M., Shrestha B.P., Style S., Harris-Fry H., Beard B.J., Sen A., . . . Costello A. Impact on birth weight and child growth of participatory learning and action women's groups with and without transfers of food or cash during pregnancy: Findings of the Low Birth Weight South Asia cluster-randomised controlled trial (LBWSAT) in Nepal. PLoS ONE 2018;13:e0194064.

**IMPRINT**

114. Taneja S., Upadhyay R.P., Chowdhury R., Kurpad A.V., Bhardwaj H., Kumar T., . . . Bhandari N. Impact of nutritional interventions among lactating mothers on the growth of their infants in the first 6 months of life: A randomized controlled trial in Delhi, India. American Journal of Clinical Nutrition 2021;113:884-94.

**Prospera**

115. Mejia-Rodriguez F., Quezada-Sanchez A.D., Gomez-Humaran I.M., Garcia-Feregrino R., Garcia-Guerra A., Fernandez-Gaxiola A.C., Neufeld L.M. Differential effects of three nutritional supplements on the nutrient intake of pregnant women enrolled in a conditional cash transfer program in Mexico: A cluster randomized trial. Nutrients 2022;14.

116. Neufeld L.M., Garcia-Guerra A., Quezada A.D., Theodore F., Bonvecchio Arenas A., Islas C.D., . . . Habicht J.P. A fortified food can be replaced by micronutrient supplements for distribution in a Mexican social protection program based on results of a cluster-randomized trial and costing analysis. Journal of Nutrition 2019;149:2302S-9S.

117. Young S.L., Blanco I., Hernandez-Cordero S., Pelto G.H., Neufeld L.M. Organoleptic properties, ease of use, and perceived health effects are determinants of acceptability of micronutrient supplements among poor Mexican women. Journal of Nutrition 2010;140:605-11.

**Senegal**

118. Cisse A.S., Dossou N., Ndiaye M., Gueye A.L., Diop el H.I., Diaham B., . . . Wade S. Stable isotope aided evaluation of community nutrition program: Effect of food supplementation schemes on maternal and infant nutritional status. Food & Nutrition Bulletin 2002;23:169-73.

**Oportunidades**

119. Barber S.L., Gertler P.J. The impact of Mexico's conditional cash transfer programme, Oportunidades, on birthweight. Tropical Medicine & International Health 2008;13:1405-14.

120. Leroy J.L., Garcia-Guerra A., Garcia R., Dominguez C., Rivera J., Neufeld L.M. The Oportunidades program increases the linear growth of children enrolled at young ages in urban Mexico. Journal of Nutrition 2008;138:793-8.

**PROCOMIDA**

121. Leroy J.L., Olney D.K., Ruel M.T. Procomida, a food-assisted maternal and child health and nutrition program, contributes to postpartum weight retention in Guatemala: A cluster-randomized controlled intervention trial. Journal of Nutrition 2019;149:2219-27.

122. Olney D.K., Leroy J., Bliznashka L., Ruel M.T. Procomida, a food-assisted maternal and child health and nutrition program, reduces child stunting in Guatemala: A cluster-randomized controlled intervention trial. Journal of Nutrition 2018;148:1493-505.

**WINGS**

123. Taneja S., Chowdhury R., Dhabhai N., Upadhyay R.P., Mazumder S., Sharma S., . . . Wings Study Group. Impact of a package of health, nutrition, psychosocial support, and wash interventions delivered during preconception, pregnancy, and early childhood periods on birth outcomes and on linear growth at 24 months of age: Factorial, individually randomised controlled trial. British Medical Journal 2022;379:e072046.

**INCAP**

124. Barrett D.E., Radke-Yarrow M. Effects of nutritional supplementation on children's responses to novel, frustrating, and competitive situations. American Journal of Clinical Nutrition 1985;42:102-20.

125. Delgado H.L., Valverde V.E., Martorell R., Klein R.E. Relationship of maternal and infant nutrition to infant growth. Early Human Development 1982;6:273-86.

126. Delgado H., Martorell R., Brineman E., Klein R.E. Nutrition and length of gestation. Nutrition Research 1982;2:117-26.

127. Lechtig A., Habicht J.P., Delgado H., Klein R.E., Yarbrough C., Martorell R. Effect of food supplementation during pregnancy on birthweight. Pediatrics 1975;56:508-20.

128. Martorell R. History and design of the INCAP longitudinal study (1969-1977) and its impact in early childhood. Food & Nutrition Bulletin 2020;41:S8-S22.

129. Webb A.L., Conlisk A.J., Barnhart H.X., Martorell R., Grajeda R., Stein A.D. Maternal and childhood nutrition and later blood pressure levels in young Guatemalan adults. International Journal of Epidemiology 2005;34:898-904.

**MINIMat**

130. Ekstrom E.C., Lindstrom E., Raqib R., El Arifeen S., Basu S., Brismar K., . . . Persson L.A. Effects of prenatal micronutrient and early food supplementation on metabolic status of the offspring at 4.5 years of age. The MINIMat randomized trial in rural Bangladesh. International Journal of Epidemiology 2016;45:1656-67.

131. Eneroth H., El Arifeen S., Persson L.A., Lonnerdal B., Hossain M.B., Stephensen C.B., Ekstrom E.C. Maternal multiple micronutrient supplementation has limited impact on micronutrient status of Bangladeshi infants compared with standard iron and folic acid supplementation. Journal of Nutrition 2010;140:618-24.

132. Hawkesworth S., Wagatsuma Y., Kahn A.I., Hawlader M.D., Fulford A.J., Arifeen S.E., . . . Moore S.E. Combined food and micronutrient supplements during pregnancy have limited impact on child blood pressure and kidney function in rural Bangladesh. Journal of Nutrition 2013;143:728-34.

133. Islam Khan A. Effects of pre- and postnatal nutrition interventions on child growth and body composition: The MINIMat trial in rural Bangladesh. Global Health Action 2013;6:22476.

134. Kallioinen M., Ekstrom E.C., Khan A.I., Lindstrom E., Persson L.A., Rahman A., Selling K.E. Prenatal early food and multiple micronutrient supplementation trial reduced infant mortality in Bangladesh, but did not influence morbidity. Acta Paediatrica 2017;106:1979-86.

135. Khan A.I., Kabir I., Ekstrom E.C., Asling-Monemi K., Alam D.S., Frongillo E.A., . . . Persson L.A. Effects of prenatal food and micronutrient supplementation on child growth from birth to 54 months of age: A randomized trial in Bangladesh. Nutrition Journal 2011;10:134.

136. Khan A.I., Kabir I., Hawkesworth S., Ekstrom E.C., Arifeen S., Frongillo E.A., Persson L.A. Early invitation to food and/or multiple micronutrient supplementation in pregnancy does not affect body composition in offspring at 54 months: Follow-up of the MINIMat randomised trial, Bangladesh. Maternal & Child Nutrition 2015;11:385-97.

137. Khan A.I., Kabir I., Eneroth H., El Arifeen S., Ekstrom E.C., Frongillo E.A., Persson L.A. Effect of a randomised exclusive breastfeeding counselling intervention nested into the MINIMat prenatal nutrition trial in Bangladesh. Acta Paediatrica 2017;106:49-54.

138. Persson L.A., Arifeen S., Ekstrom E.C., Rasmussen K.M., Frongillo E.A., Yunus M., MINIMat Study Team. Effects of prenatal micronutrient and early food supplementation on maternal hemoglobin, birth weight, and infant mortality among children in Bangladesh: The MINIMat randomized trial. Journal of the American Medical Association 2012;307:2050-9.

139. Shaheen R., Streatfield P.K., Naved R.T., Lindholm L., Persson L.A. Equity in adherence to and effect of prenatal food and micronutrient supplementation on child mortality: Results from the MINIMat randomized trial, Bangladesh. BMC Public Health 2014;14:5.

140. Shaheen R., Persson L.A., Ahmed S., Streatfield P.K., Lindholm L. Cost-effectiveness of invitation to food supplementation early in pregnancy combined with multiple micronutrients on infant survival: Analysis of data from MINIMat randomized trial, Bangladesh. BMC Pregnancy & Childbirth 2015;15:125.

141. Siddiqua T.J., Roy A.K., Akhtar E., Haq M.A., Wagatsuma Y., Ekstrom E.C., . . . Raqib R. Prenatal nutrition supplementation and growth biomarkers in preadolescent Bangladeshi children: A birth cohort study. Maternal & Child Nutrition 2022;18:e13266.

142. Svefors P., Selling K.E., Shaheen R., Khan A.I., Persson L.A., Lindholm L. Cost-effectiveness of prenatal food and micronutrient interventions on under-five mortality and stunting: Analysis of data from the MINIMat randomized trial, Bangladesh. PLoS ONE 2018;13:e0191260.

143. Tofail F., Persson L.A., El Arifeen S., Hamadani J.D., Mehrin F., Ridout D., . . . Grantham-McGregor S.M. Effects of prenatal food and micronutrient supplementation on infant development: A randomized trial from the maternal and infant nutrition interventions, Matlab (MINIMat) study. Americal Journal of Clinical Nutrition 2008;87:704-11.

144. Ziaei S., Rahman A., Raqib R., Lonnerdal B., Ekstrom E.C. A prenatal multiple micronutrient supplement produces higher maternal vitamin B-12 concentrations and similar folate, ferritin, and zinc concentrations as the standard 60-mg iron plus 400-mug folic acid supplement in rural Bangladeshi women. Journal of Nutrition 2016;146:2520-9.

**South Africa Ross**

145. Ross S.M., Nel E., Naeye R.L. Differing effects of low and high bulk maternal dietary supplements during pregnancy. Early Human Development 1985;10:295-302.

**Mamachiponde**

146. Callaghan-Gillespie M., Schaffner A.A., Garcia P., Fry J., Eckert R., Malek S., . . . Papathakis P.C. Trial of ready-to-use supplemental food and corn-soy blend in pregnant Malawian women with moderate malnutrition: A randomized controlled clinical trial. American Journal of Clinical Nutrition 2017;106:1062-9.

147. Glosz C.M., Schaffner A.A., Reaves S.K., Manary M.J., Papathakis P.C. Effect of nutritional interventions on micronutrient status in pregnant Malawian women with moderate malnutrition: A randomized, controlled trial. Nutrients 2018;10:07.

**Sierra Leone RUSF**

148. Hendrixson D.T., Smith K., Lasowski P., Callaghan-Gillespie M., Weber J., Papathakis P., . . . Manary M.J. A novel intervention combining supplementary food and infection control measures to improve birth outcomes in undernourished pregnant women in Sierra Leone: A randomized, controlled clinical effectiveness trial. PLOS Medicine 2021;18:1-18.

149. Koroma A.S., Ellie M., Bangura K., Iversen P.O., Hendrixson D.T., Stephenson K., Manary M.J. Supplementary feeding and infection control in pregnant adolescents-a secondary analysis of a randomized trial among malnourished women in Sierra Leone. Maternal & Child Nutrition 2023;19:e13456.
